# TrkC, a novel prognostic marker, induces and maintains cell survival and metastatic dissemination of Ewing sarcoma by inhibiting EWSR1-FLI1 degradation

**DOI:** 10.1038/s41419-022-05275-w

**Published:** 2022-09-28

**Authors:** Min Soo Kim, Won Sung Lee, Hanki Lee, Wook Jin

**Affiliations:** 1 Laboratory of Molecular Disease and Cell Regulation, Department of Biochemistry, School of Medicine, Gachon University, Incheon, 21999 Republic of Korea; 2 Graduate School of Interdisciplinary Program of Biomodulation, Myongji University, Yongin, Gyeonggi-do 17058 Republic of Korea

**Keywords:** Sarcoma, Tumour-suppressor proteins, Metastasis

## Abstract

Upregulation of EWSR1-FLI1 expression has been associated with invasiveness, induced cell survival, metastatic dissemination, and acquisition of self-renewal traits in Ewing sarcoma (ES). Although existing evidence implies that TrkC expression is linked to the pathogenesis of other cancer types, its role and the mechanism behind its correlation with EWSR1-FLI1 in the pathogenesis of ES remain unclear. In this study, we uncovered a novel physiological role of TrkC as a key regulator of EWSR1-FLI1 involved in the survival and metastatic dissemination of ES. TrkC was observed to be frequently overexpressed in human metastatic ES cells in vitro and in vivo, facilitating enhanced survival, tumorigenicity, and metastasis of ES cells. TrkC-mediated metastasis of ES cells was induced by the inhibition of the proteasomal degradation of EWSR1-FLI1 via the TrkC/EWSR1-FLI1 complex, which subsequently enabled the induction of the target proteins, EGR2 and NKX2.2. Moreover, TrkC significantly inhibited tumor suppressor activity of TGF-β through reduction of the mRNA expression of one of its receptors, TGFBR2 via TrkC-induced stabilization of EWSR1-FLI1. Furthermore, loss of TrkC expression inhibited tumor growth and metastasis in experimental mouse models. This study is the first to report the involvement and functional role of TrkC in the pathogenesis of ES, suggesting important implications for understanding the alterations of TrkC in Ewing tumors.

## Introduction

Ewing sarcoma (ES) is a rare and highly aggressive bone-associated tumor that affects both children and young adults. Although ES often responds well to chemotherapy, the 5-year overall survival (OS) rate of patients diagnosed with ES has been reported as 52–75% [1], with an approximately two-fold difference in response rates between adults and elderly patients (42% vs. 19%) [1, 2]. Meanwhile, chemotherapy has been reported to increase the OS rate by six months relative to the non-chemotherapy group [3], the five year OS rate of patients with metastatic ES remains less than 30% [4].

EWSR1-FLI1 has been identified as the central player in the pathogenesis of ES and is detected in 85–90% of patients diagnosed with ES [5]. EWSR1-FLI1 promotes cellular transformation by inducing or repressing target genes [6, 7]. EWSR1-FLI1 also promotes tumorigenicity through modulation of stemness in ES cancer stem cells (CSCs). Expression of EWSR1-FLI1 in mouse mesenchymal progenitor cells is sufficient to induce the transformation and development of ES-like tumors in vivo [8, 9]. Moreover, EWSR1-FLI1 induces the expression of the embryonic stem cell (ESC)-specific genes *OCT4*, *SOX2*, and *NANOG* in human pediatric mesenchymal stem cells (hpMSCs). hpMSCs expressing EWSR1-FLI1 have the ability to generate a subpopulation displaying CSC features in Ewing tumors [10]. Furthermore, the pathogenesis of ES cells depends on the expression of EWSR1-FLI1. However, EWSR1-FLI1 is a target of the ubiquitin system, which is primarily degraded by the proteasome via polyubiquitination at a single lysine residue [11]. A recent study demonstrated that Tripartite Motif Containing 8 (TRIM8), an E3 ligase, regulates cell growth of ES cells by reducing EWSR1-FLI1 stability [12]. However, the underlying mechanism by which EWSR1-FLI1 maintains its expression in ES cells is largely unknown.

TrkC has been identified as a prominent multi-functional factor in cancer. Its association with cancer has been widely attributed to its involvement in the regulation of angiogenesis [13], tumorigenesis, and metastasis of congenital fibrosarcoma, breast cancer, and colorectal cancer [14–16]. Overexpression of TrkC activates IL6/JAK2/STAT3 and PI3K/AKT/mTOR signaling or suppresses the bone morphogenetic protein (BMP) signaling, thereby contributing to the epithelial–mesenchymal transition (EMT)-related progression and cell survival in breast, leukemia, and colorectal cancers [15–18]. Although the role of TrkC has been identified in other cancer types, its contribution to tumorigenesis and metastasis of ES remains uninvestigated.

In this study, we, therefore, for the first time, identified the role of TrkC in tumorigenesis and metastasis of ES. We further revealed the involvement of a novel functional link between EWSR1-FLI1 and TrkC in ES pathogenesis that acts through the regulation of transforming growth factor beta type2 receptor (TGFBR2).

## Results

### Elevated TrkC expression in ES

**Fig. 1:**
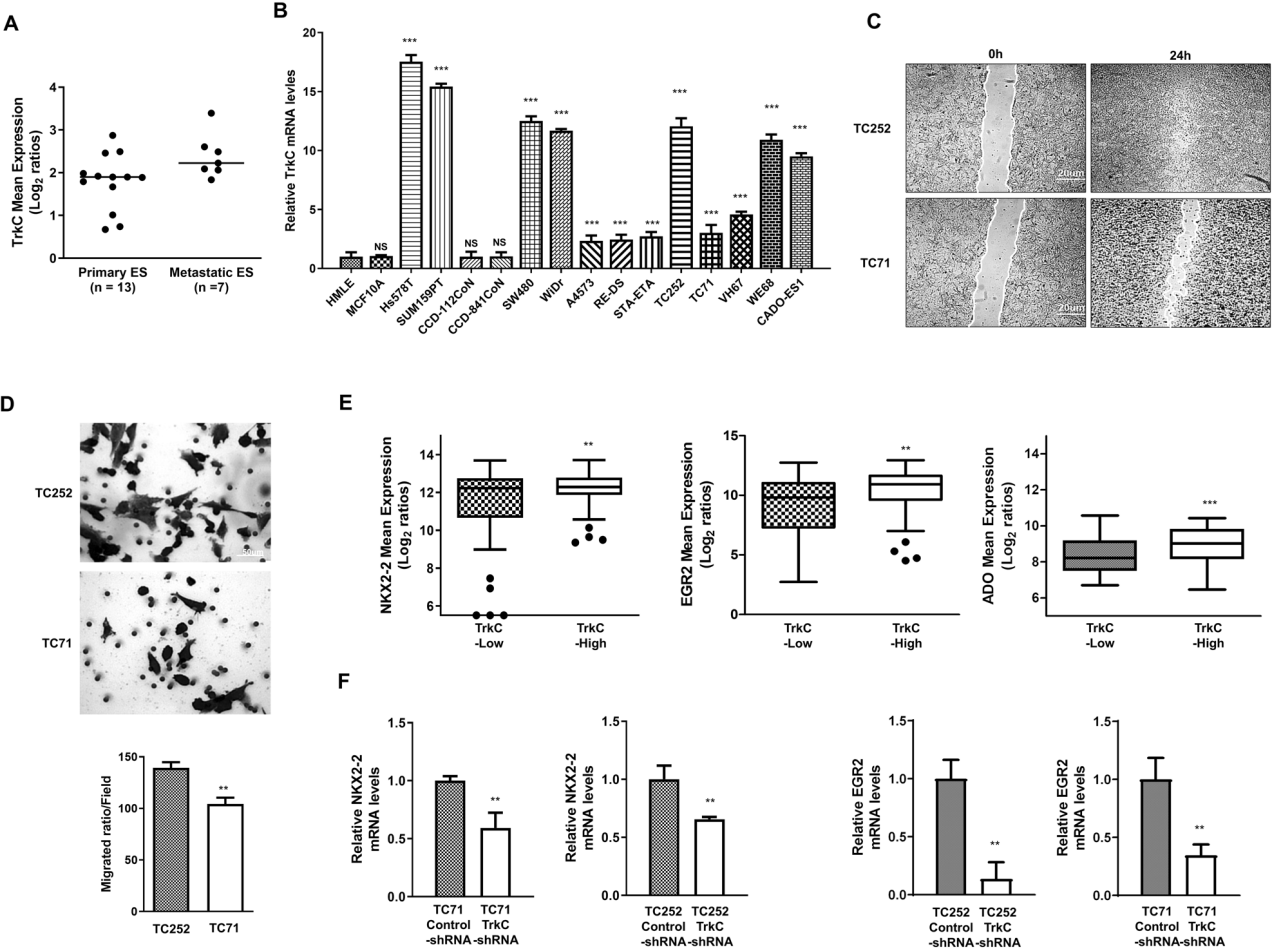
TrkC expression is correlated with the pathogenesis of ES. **A** Scatter plots present the expression of TrkC in primary and metastatic ES. The TrkC level of independent primary and metastatic tumor samples of ES patients was extracted from the Scotlandi microarray dataset (GSE12102) and averaged for each tumor. Statistical significance was determined through *t* test; *P* value < 0.05 (**). **B** The expression of the mRNA encoding TrkC in ES, breast (HS578T, SUM159PT), and colorectal (SW480, WiDr) cancer cell lines relative to its normal (human mammary epithelial cell (HMLE), MCF10A, CCD-112CoN, CCD-841 CoN cell), as determined by quantitative RT-PCR. The 18 S was used for loading control. *n* = 3. *P* values < 0.001 (***); *t* test. NS: Not significant. **C** Wound-healing assay of TC252 and TC71 ES cells. Wound closures were photographed at 0 and 18 h after wounding. *n* = 3. **D** The transwell assay of in TC252 and TC71 ES cells. Migrated cells were photographed after analysis. *n* = 3. *P* values < 0.05 (**); *t* test. **E** Box-and-whisker (Tukey) plots are shown for the expression of NKX2.2, EGR2, and ADO in 117 primary tumors of ES patients from the Postel-Vinay dataset (GSE34620) after being divided into high and low TrkC expressers. *P* values < 0.05 (**) or *P* values < 0.001 (***); *t* test. **F** The expression of the mRNA encoding NKX2.2 and EGR2 in TC252 and TC71 control-shRNA or TrkC-shRNA cells. The 18S was used as a loading control. *n* = 3. *P* values < 0.05 (**); *t* test.

Although TrkC expression is linked to certain types of cancer, it has not been well characterized in human ES. In this study, we evaluated its potential involvement in the pathogenesis of ES using a public microarray dataset (GSE12102), independently collected from primary and metastatic tumor samples from patients with ES [19]. TrkC levels were remarkably higher in patients with metastatic ES than those in patients with primary ES (Fig. 1A), however, they remained similar to those observed in patients with relapsed ES (Fig. S1). To validate the association of *TrkC* expression in ES in vitro, we next examined *TrkC* expression in human ES cell lines. *TrkC* was markedly induced in ES (A4753, RE-DS, STA-ETA, TC252, TC71, VH67, WE68, and CADO-ES1), breast (Hs578T and SUM159), and colorectal (SW480 and WiDr) cancer cell lines compared to its normal (human mammary epithelial cell (HMLE), MCF10A, CCD-112CoN, and CCD-841 CoN), which do not express *TrkC* (Fig. 1B). To determine the association between the metastatic ability of ES cells with TrkC expression, we conducted a wound-healing and migration assay using TC252 cells with high TrkC expression and TC71 cells with a relatively lower level of TrkC compared to that in TC252 cells. Interestingly, TC252 cells presented significantly higher migration ability than the TC71 cells (Figs. 1C, D, S2), indicating that TrkC may be correlated with the metastatic ability of ES.

EGR2, ADO, NKX2-2, and TARDBP have been identified as new molecular signatures associated with the proliferation and metastatic ability of ES [20–26]. Therefore, we aimed to examine the correlation between the expression of these signatures with that of TrkC in 117 patients with independent primary ES using GSE34620 [21]. TrkC expression was observed to positively correlate with that of *NKX2-2*, *EGR2*, and *ADO*, revealed through a marked increase in levels of these markers in patients with overexpressed TrkC than in those with relatively low TrkC expression (Fig. 1E). *TARDBP* expression, however, did not alter with changes inTrkC expression in patients (Fig. S3). Therefore, we hypothesized that TrkC contributes to tumorigenicity and metastasis in human ES. To test this hypothesis, we introduced stably expressing TrkC-shRNAs into selected TC252 and TC71 cells, which resulted in a 50% and 58% reduction of TrkC expression, respectively (Fig. S4). This observation was accompanied by TrkC-shRNA-mediated downregulation of the expression of *NKX2-2* and *EGR2* relative to the respective control cells (Fig. 1F). Combinedly, these results indicated that TrkC may be a key mediator in ES tumorigenicity and acts through the upregulation of selected tumor-associated molecular markers, *NKX2-2* and *EGR2*.

### TrkC was required for the acquisition of metastatic ability of ES

**Fig. 2:**
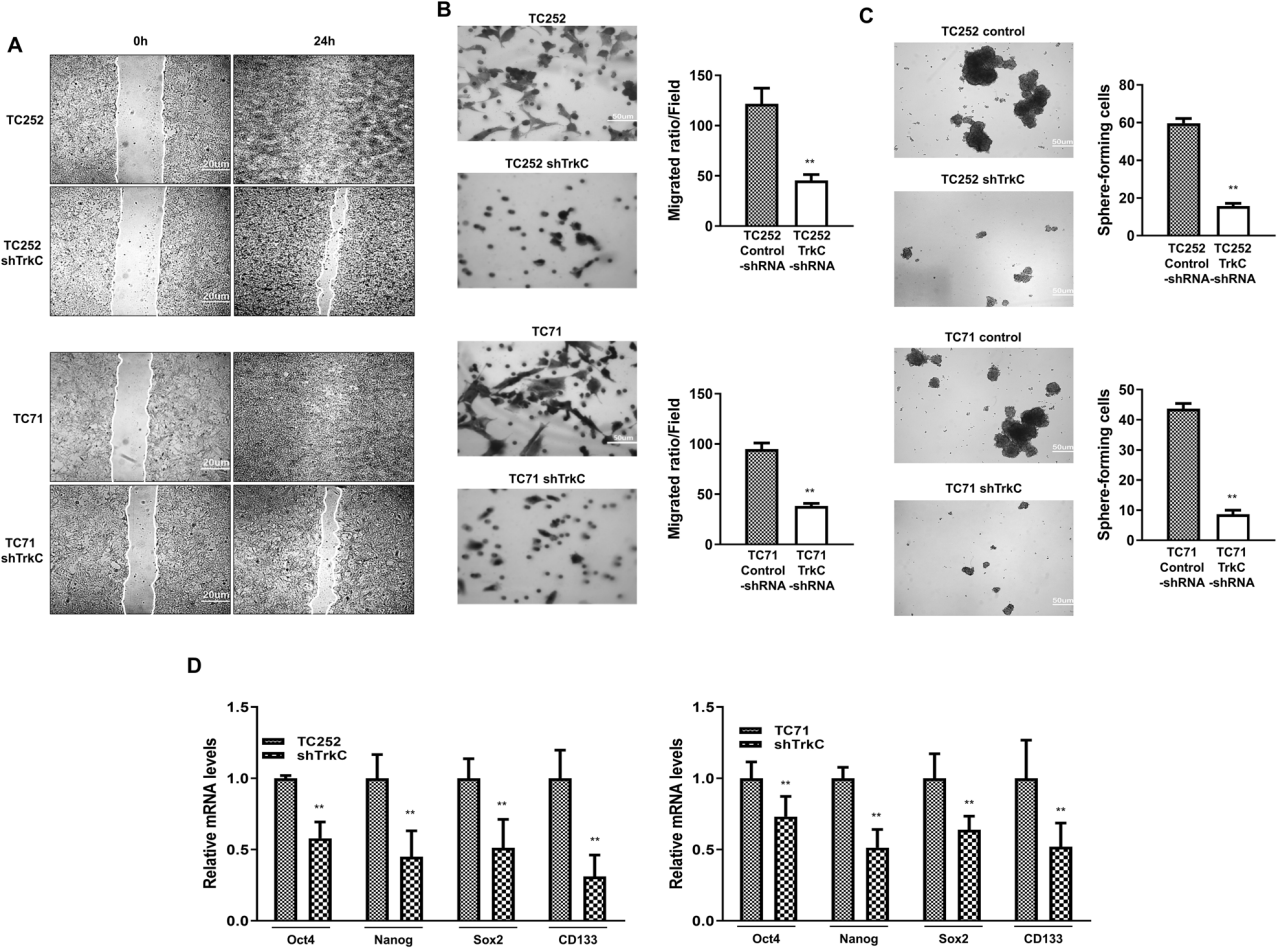
TrkC is essential for the induction of metastatic ability of ES cells. **A** Wound-healing assay of TC252 and TC71 control-shRNA and TrkC-shRNA cells. Wound closures were photographed at 0 and 24 h after wound generation. *n* = 3. **B** The migration assay of TC252 and TC71 control-shRNA and TrkC-shRNA cells. Migrated cells were photographed after analysis. *P* values < 0.05 (**); *t* test. *n* = 3. **C** Sphere formation assay of TC252 and TC71 control-shRNA and TrkC-shRNA cells. Sphere formations were photographed. *n* = 3. *P* values < 0.05 (**); *t* test. **D** Quantitative RT-PCR analysis of Oct4, Nanog, Sox2, and CD133 expression in TC252 and TC71 control-shRNA and TrkC-shRNA cells. The 18S was used for loading control. *n* = 3. *P* values < 0.05 (**); *t* test.

To examine the effect of TrkC in tumorigenesis of ES, we assessed the motility of the ES cells. TC252 and TC71 control cells showed significantly increased cell migration as compared to the respective TrkC-shRNA-engineered cells (Figs. 2A, B, S5).

Malignancy affects the morphogenesis and acquisition of the self-renewing trait of CSCs [27, 28]. In our study, we examined whether TrkC affected cell proliferation at a clonal density associated with the mesenchymal stem cell population [29, 30]. The sphere-forming cell population in the control TC252 and TC71 cells were 3.8- and 5-folds of the respective TrkC-shRNA cells (Fig. 2C). Additionally, TrkC expression was observed to increase the expression of CSC markers, Oct4, Nanog, Sox2, and CD133 (Fig. 2D). CD133 is associated with strong chemoresistance in ES [31], and maintenance of CSCs [32]. The above results indicated that TrkC expression markedly increased the acquisition of the CSC traits by enriching sphere-forming cells.

### TrkC inhibited apoptosis of ES cells

**Fig. 3:**
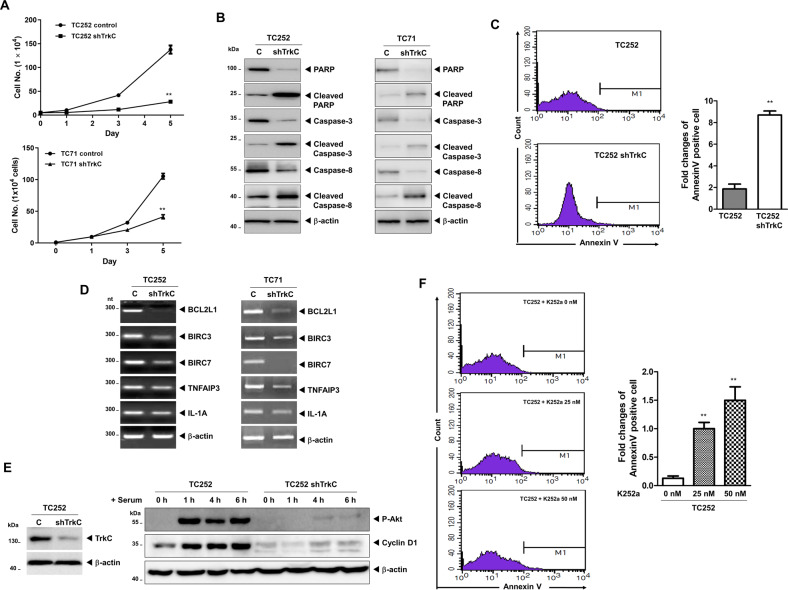
Suppression of TrkC reduces the survival of ES cells. **A** Quantification of cell growth of TC252 and TC71 control-shRNA and TrkC-shRNA cells. Each data point represents the mean of cells counted in three dishes. *n* = 3. *P* values < 0.05 (**); *t* test. **B** Immunoblot analysis of cleaved caspase-3, caspase-8, and PARP or total proteins (caspase-3, caspase-8, and PARP) in TC252 and TC71 control-shRNA and TrkC-shRNA cells. β-actin was used as a loading control. **C** Representative FACS histograms showing the percentage of apoptotic TC252 control-shRNA and TrkC-shRNA cells, as determined by binding of FITC-conjugated Annexin V. The bar graph shows the mean number of Annexin V-positive cells, expressed as a percentage of total cells. *n* = 3. *P* values < 0.05 (**); *t* test. **D** RT-PCR analysis of anti-apoptotic genes (BLC2L1, BIRC3, BIRC7, TNFAIP3, and IL-1A) in TC252 and TC71 control-shRNA and TrkC-shRNA cells. β-actin was used as a loading control. **E** Immunoblot analysis of phospho-AKT and cyclin D1 in TC252 control-shRNA and TrkC-shRNA cells. After 16 h of serum starvation, these cells were treated with 10% FBS for 0, 1, 4, and 6 h. β-actin was used as a loading control. **F** Representative FACS histograms showing the percentage of apoptotic control-shRNA and TrkC-shRNA cells in anchorage-independent conditions, as determined by binding of FITC-conjugated Annexin V. The bar graph shows the mean number of Annexin V-positive cells, expressed as a percentage of total cells. *n* = 3. *P* values < 0.05 (**); *t* test.

To determine whether TrkC is essential for the survival of ES cells. TC252 and TC71 TrkC-shRNA cells showed significantly reduced proliferation compared to the respective control cells (Fig. 3A). We also measured the anchorage-independent survival of ES cells. Metastatic cancer cells complete various metastatic steps by overcoming anoikis [33], and the anchorage-independent growth of ES is more closely related to primary tumors in terms of cell morphology, cell–cell junctions, and cell proliferation [34]. TC252 and TC71 TrkC-shRNA cells formed large spheroid aggregates and significantly increased the cell population compared to the respective control cells (Fig. S6A). In addition, the loss of TrkC demonstrated a marked increase in the levels of cleaved PARP and activated caspases-3 and -8 relative to the respective control cells (Fig. 3B). To further characterize the role of TrkC in the survival of ES cells, we assessed the translocation of phosphatidylserine using annexin V. The number of early apoptotic cells increased 4.7-fold in TC252 TrkC-shRNA cells compared to that in control cells (Fig. 3C). This result indicates that TrkC increases the survival of ES cells by blocking apoptosis. Members of the inhibitor of apoptosis protein family contribute to tumor cell survival, chemoresistance, disease progression, and poor prognosis [35, 36]. In agreement with these results, knockdown of TrkC significantly decreased the mRNA expression of anti-apoptotic genes (Fig. 3D).

Phosphatidylinositol-3 kinase (PI3K)/protein kinase B (Akt) pathway is a critical factor for the proliferation and metastasis of ES cells and acts by inducing the expression of cyclin D1, EGR2, and NKX2-2 [34, 37, 38]. Based on our above observations, we speculated that TrkC modulates the expression of cyclin D1, EGR2, and NKX2-2 in ES cells by activating the PI3K/AKT pathway. Interestingly, the levels of phosphorylated AKT and cyclin D1 in TC252 and TC71 TrkC-shRNA cells were significantly lower than those in the respective control cells (Figs. 3E, S7). To determine whether tyrosine kinase activity of TrkC is essential for the survival of ES cells, TC252 and TC71 cells were treated with a Trk inhibitor, K252 The proliferation rate of TrK inhibited cells was significantly lower than that of the untreated cells (Fig. S6B). TC252 and TC71 cells with uncompromised TrK readily proliferated as large spheroid aggregates in suspension, similar to the spheroids formed by ES cells grown in suspension described elsewhere [34]. In contrast, cell proliferation of these cells was significantly reduced under K252a treatment regardless of the concentration, thereby preventing the formation of large spheroid aggregates in suspension (Fig. S6C). To further characterize the inhibition of apoptosis by TrkC, we assessed the translocation of phosphatidylserine using annexin V. K252a treatment dramatically increased the number of apoptotic TC252 cells in a dose-dependent manner (Fig. 3F). These results indicated that the expression-mediated induction of tyrosine kinase activity of TrkC is critical for the survival and tumorigenicity of ES cells.

### The effect of TrkC on tumorigenicity and metastasis of ES

**Fig. 4:**
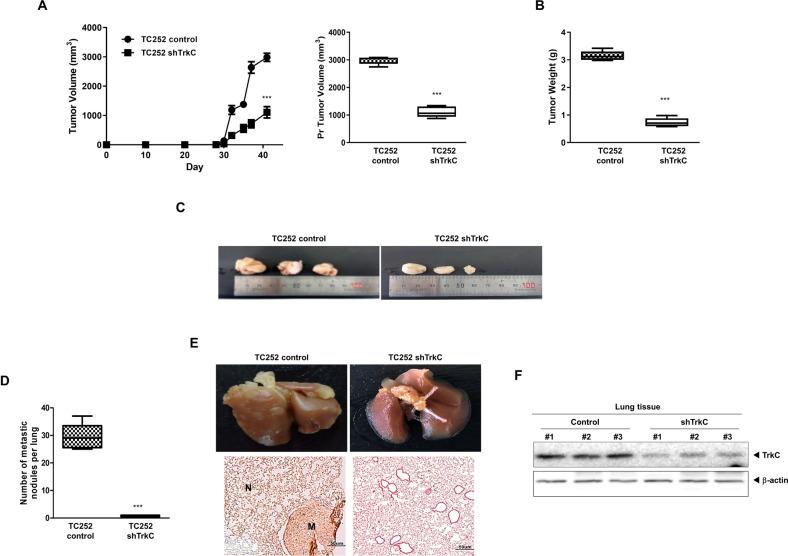
TrkC induced the primary tumor formation and metastatic dissemination of ES in vivo. **A** Primary tumor formation by TC252 cells expressing either the control-shRNA or TrkC-shRNA. A total of 1.0 × 10^6^ cells were injected subcutaneously into the flanks of mice (*n* = 7). *P* values < 0.001 (***); *t* test. **B** The tumor weight from mice carrying TC252 control-shRNA and TrkC-shRNA cells was measured and presented as mean ± SEM (*n* = 7). *P* values < 0.001 (***); *t* test. **C** Representative images of tumors from mice carrying TC252 control-shRNA and TrkC-shRNA cells. **D** Representative images of lung metastasis by TC252 control-shRNA or TC252 TrkC-shRNA cells (*n* = 7). *P* values < 0.001 (***); *t* test. **E** The total number (upper) and histological analysis (lower) of metastatic lung nodules from each mouse harboring TC252 control-shRNA or TrkC-shRNA. Representative hematoxylin and eosin (H&E) staining in the sections of the lungs from individual mice expressing either TC252 control-shRNA or TrkC-shRNA. N, Normal lung tissue; M, metastatic nodule. **F** Immunoblot analysis of TrkC in tumor cells recovered from the lungs of individual mice expressing either TC252 control-shRNA or TrkC-shRNA. β-actin was used as a loading control. *n* = 3.

To determine whether the TrkC contributes to the primary tumor formation of ES cells in vivo, we implanted TrkC expressing control or TrkC-shRNA-engineered TC252 cells subcutaneously in BALB/c Nu/Nu mice and analyzed the formation of primary tumors. In the control group, subcutaneous tumor formation was observed. In contrast, suppression of TrkC significantly reduced primary tumor formation (Fig. 4A). In addition, the volume and weight of tumors originating from TC252 TrkC-shRNA cells were approximately 2- and 4.2-fold lighter than those originating from TC252 control cells, respectively (Fig. 4B, C). These results combinedly suggested that TrkC increases the ability of TC252 cells to proliferate and survive in vivo.

We further investigated whether TrkC contributed to metastatic dissemination of the tumor cells in vivo. For this purpose, TC252 control and TrkC-shRNA cells were injected into the tail vein of mice. The average number of visible metastatic nodules in the lungs of mice injected with TC252 TrkC-shRNA cells was significantly lower than those injected with the control cells (Fig. 4D, E). To exclude the possibility that the metastatic nodule formation was due to suppressed TrkC, we examined the comparative TrkC expression in the lungs of mice injected with TC252 control- or TrkC-shRNA cells. TrkC expression was significantly reduced in the lungs of mice harboring TC252 cells expressing TrkC-shRNA relative to its control (Fig. 4F). These results indicate that continued TrkC expression is required to efficiently execute and maintain metastatic dissemination and primary tumor formation in ES.

### TrkC inhibited TGF-β signaling by suppression of TGF-β type II receptor

**Fig. 5:**
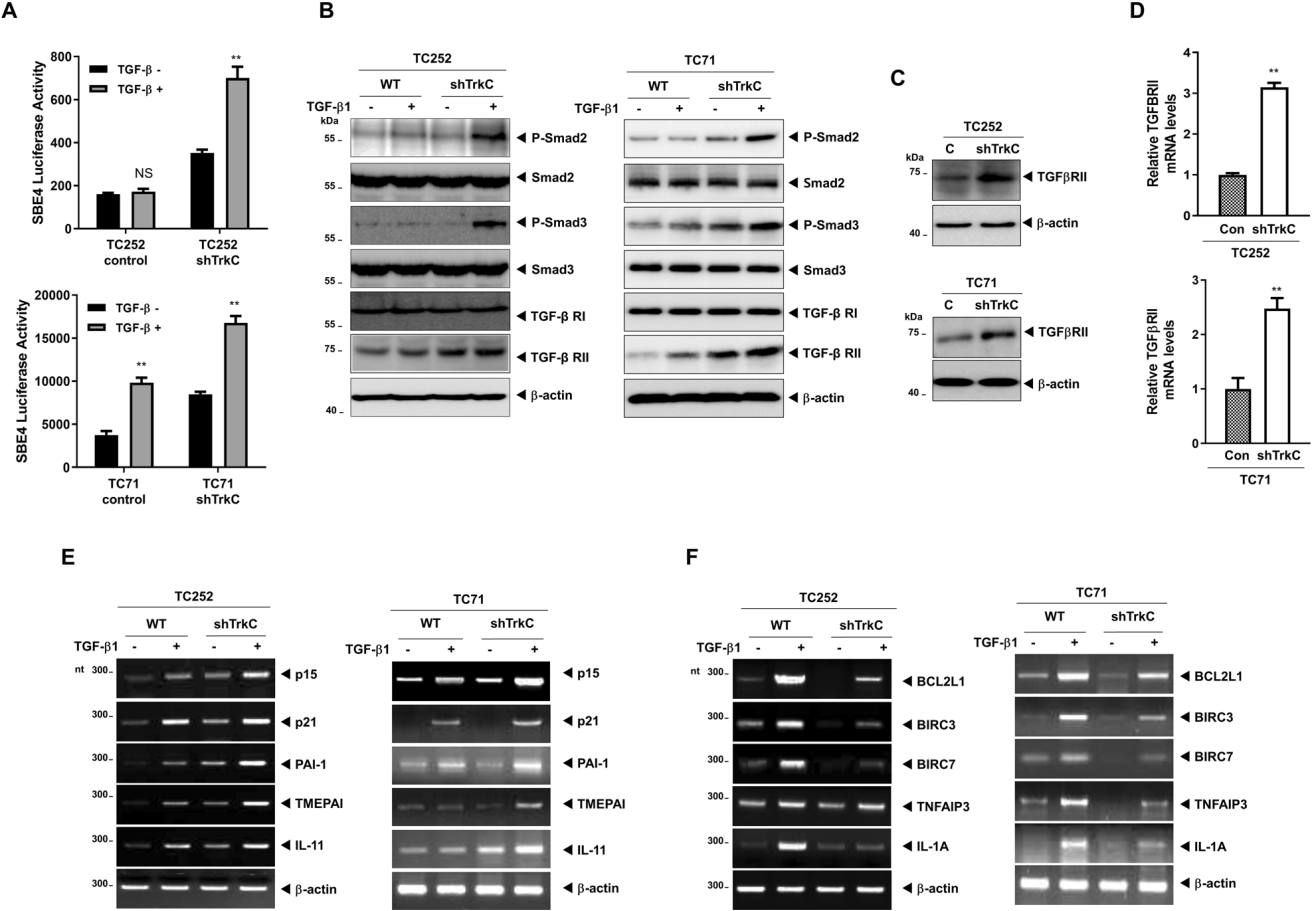
TrkC inhibited TGF-β-mediated tumor suppressor activity. **A** The activity of TGF-β1-responsive SBE luciferase reporter in TC252 and TC71 control-shRNA or TrkC-shRNA cells. Luciferase activity was measured at 24 h after treatment with TGF-β1 (5 ng/mL). *n* = 3. *P* values < 0.05 (**); *t* test, NS: Not significant. **B** Immunoblot analysis of the expression of phospho-SMAD2, phospho-SMAD3, SMAD2, SMAD3, TGF-β type I or II receptors in TC252 and TC71 control-shRNA or TrkC-shRNA cells after stimulation with TGF-β1 (5 ng/mL). β-actin was used as a loading control. **C** Immunoblot analysis of the expression of TGF-β type II receptor in TC252 and TC71 control-shRNA or TrkC-shRNA cells. β-actin was used as a loading control. **D** Quantitative RT-PCR analyses of TGF-β type II receptor in TC252 and TC71 control-shRNA or TrkC-shRNA cells. The 18S was used for loading control. *n* = 3. *P* values < 0.05 (**); *t* test. **E** RT-PCR analyses of p15^Ink4b^, p21^Cip1^, PAI1, TMEPAI, and IL-11 mRNAs in TC252 and TC71 control-shRNA or TrkC-shRNA cells after treatment with or without TGF-β1 (5 ng/mL). β-actin was used as a loading control. **F** RT-PCR analyses of BCL2L1, BIRC3, BIRC7, TNFAIP3, and IL-1A mRNAs in TC252 and TC71 control-shRNA or TrkC-shRNA cells after treatment with or without TGF-β1 (5 ng/mL). β-actin was used as a loading control.

TGF-β signaling has been reported to inhibit tumor cell plasticity of ES [39]. Therefore, we focused on the possible modulation of TGF-β signaling in ES to understand the role of TrkC. The luciferase activity of *SBE4* and *3TP* was significantly increased in TC252 and TC71 TrkC-shRNA cells relative to the respective control cells (Figs. 5A, S8), which correlated with increased activation of Smad2 and Smad3 in TC252 and TC71 TrkC-shRNA cells, respectively (Fig. 5B).

Remarkably, we found that TGFBR2 expression was significantly increased in TC252 and TC71 TrkC-shRNA cells with or without TGF-β, but the level of TGF-β type I receptor remained unaltered (Fig. 5B, C). This finding was inconsistent with the biological concept mentioned in our previous reports [40, 41] demonstrating that TGF-β signaling is inhibited by TrkC or ETV6-NTRK3 mediated by a reduction of TGFBR2 phosphorylation via complex formation of TrkC/TGFBR2. Our above observation suggested that TrkC might stimulate the survival of ES cells by downregulating TGFBR2 expression as another way to regulate TGF-β signaling. To characterize a new mechanism that suppresses TGF-β signaling, we investigated the expression status of *TGFBR2* in response to TrkC using our candidate ES cells. *TGFBR2* was strongly downregulated in TC252 and TC71 control cells relative to the respective TrkC-shRNA cells (Fig. 5D). In addition, the analysis of *TGFBR2* expression in patients diagnosed with ES (*n* = 117) using GSE34620 showed that *TGFBR2* was markedly decreased in patients with high TrkC expression than in those with relatively low TrkC expression (Fig. S9).

We found that these above results correlated with TGF-β1 target gene expression. The expression of *p15*^*Ink4b*^, *p21*^*Cip1*^, *PAI1*, *TMEPAI*, and *IL11* in TC252 and TC71 TrkC-shRNA cells stimulated with TGF-β1 were markedly increased compared to the respective control cells (Fig. 5E). Additionally, we investigated whether TrkC regulates TGF-β signaling-mediated apoptosis. Although the basal expression levels of the anti-apoptotic proteins BCL2L1, BIRC3, BIRC7, TNFAIP3, and IL-1A were increased in the control cells, these levels were further upregulated after TGF-β1 treatment in TC252 and TC71 control cells compared to their respective TrkC-shRNA cells (Fig. 5F). These results indicated that TGF-β signaling induced cellular apoptosis in ES. This effect might be directly or indirectly modulated by TrkC through the suppression of *TGFBR2*, thereby inducing tumorigenicity and metastasis of ES. However, the mechanism by which TrkC regulates *TGFBR2* expression yet remains unestablished.

### TrkC regulated the EWSR1-FLI1 signaling pathway

The EWSR1-FLI1 induces the pathogenesis of ES through the dysregulation of its target genes involved in tumor development, energy metabolism, and cancer stemness [5, 42]. Also, EWSR1-FLI1 targets the tumor suppressor gene *TGFBR2* by reducing its mRNA and protein levels through interaction with its promoter and reduction of TGF-β sensitivity in ES [43, 44].

**Fig. 6:**
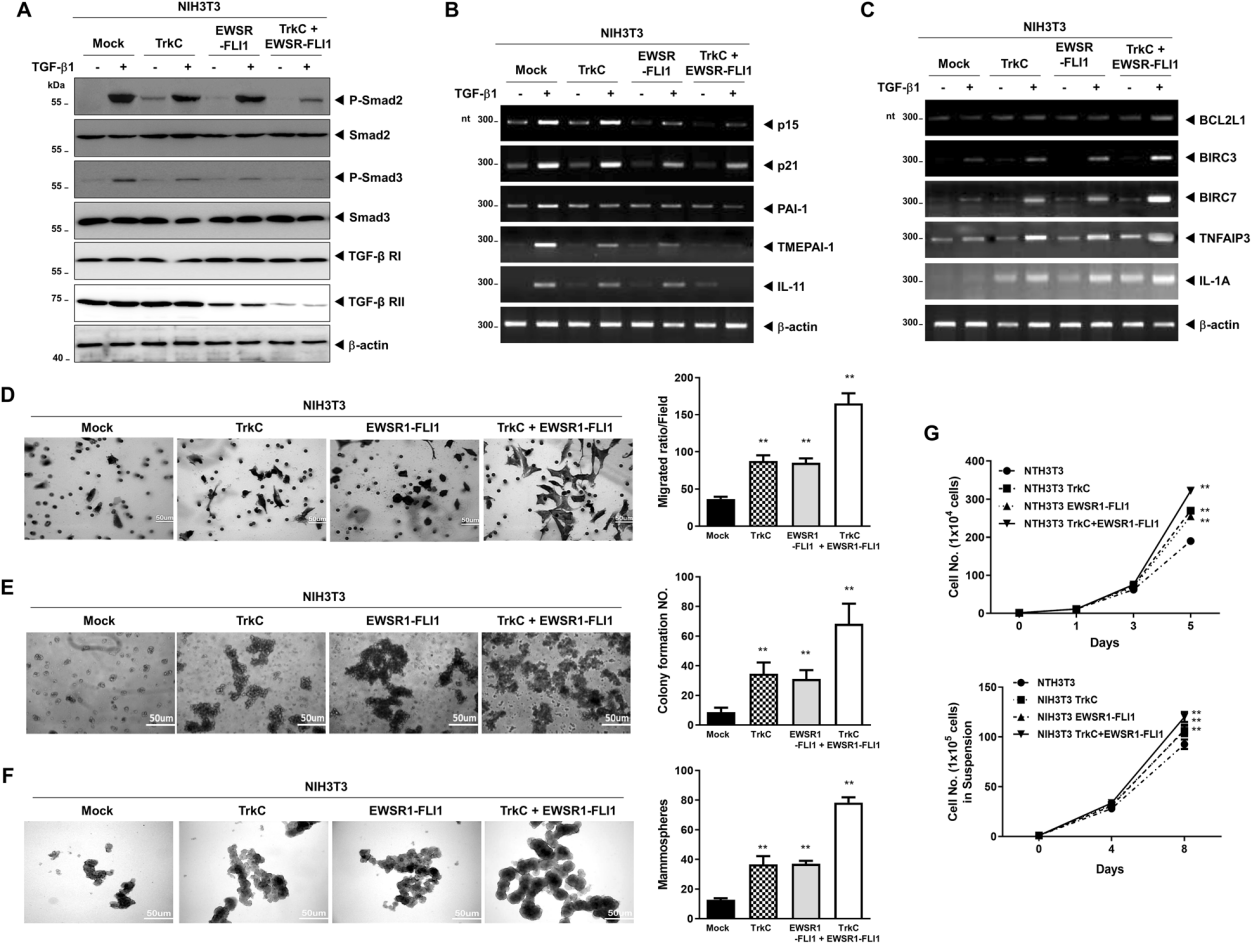
TrkC induced EWSR1-FLI1-mediated cell growth and metastatic ability in ES. **A** Immunoblot analysis of the expression of phospho-SMAD2, phospho-SMAD3, SMAD2, SMAD3, TGF-β type I or II receptors in NIH3T3 cells expressing control, TrkC, EWSR1-FLI1, or TrkC/EWSR1-FLI1 after stimulation with TGF-β1 (5 ng/mL). β-actin was used as a loading control. **B** RT-PCR analyses of p15^Ink4b^, p21^Cip1^, PAI1, TMEPAI, and IL-11 mRNAs in NIH3T3 cells expressing control, TrkC, EWSR1-FLI1, or TrkC/EWSR1-FLI1 after treatment with or without TGF-β1 (5 ng/mL). β-actin was used as a loading control. **C** RT-PCR analyses of BCL2L1, BIRC3, BIRC7, TNFAIP3, and IL-1A mRNAs in NIH3T3 cells expressing control, TrkC, EWSR1-FLI1, or TrkC/EWSR1-FLI1 after treatment with or without TGF-β1 (5 ng/mL). β-actin was used as a loading control. **D** The migration assay of NIH3T3 cells expressing control, TrkC, EWSR1-FLI1, or TrkC/EWSR1-FLI1. Migrated cells were photographed after analysis. *n* = 3. *P* values < 0.05 (**); *t* test. **E** The colony formation assay of NIH3T3 cells expressing control, TrkC, EWSR1-FLI1, or TrkC/EWSR1-FLI1. Migrated cells were photographed after analysis. *n* = 3. *P* values < 0.05 (**); *t* test. **F** Sphere formation assay of NIH3T3 cells expressing control, TrkC, EWSR1-FLI1, or TrkC/EWSR1-FLI1. Sphere formations were photographed. *n* = 3. *P* values < 0.05 (**); *t* test. **G** Population doubling of NIH3T3 cells expressing control, TrkC, EWSR1-FLI1, or TrkC/EWSR1-FLI1. Each data point represents the mean of cells counted in three dishes. *n* = 3. **NIH3T3 versus NIH3T3 TrkC, EWSR1-FLI1, and TrkC/EWSR1-FLI1 cells, *P* values < 0.05; *t* test.

Based on this evidence, we first examined the effects of TrkC on the EWSR1-FLI1-mediated suppression of TGF-β sensitivity in ES cells. NIH3T3 TrkC and EWSR1-FLI1 cells strongly decreased the luciferase activity of *SBE4* and *3TP*, and these reductions were further induced by TrkC-EWSR1-FLI1 relative to NIH3T3 control, TrkC, or EWSR1-FLI1 cells (Fig. S10). Consistent with this finding, TrkC and EWSR1-FLI1 markedly decreased the phosphorylation of Smad2 and Smad3, respectively, which was further downregulated by TrkC/EWSR1-FLI1, overall positively correlating with TGFBR2 expression. The level of TGFBR2 was significantly decreased by EWSR1-FLI1 but not TrkC. Interestingly, this level further decreased in response to TrkC/EWSR1-FLI1 (Fig. 6A), indicating that a joint cellular signaling mechanism of TrkC and EWSR1-FLI1 may be essential to induce and maintain the pathogenesis of ES.

We found that the above observations correlated with the effects of TrkC and EWSR1-FLI1 on the expression of TGF-β1 target genes and the inhibition of TGF-β-induced cell death in ES. The levels of TGF-β1 target genes post-TGF-β1 treatment decreased significantly in EWSR1-FLI1 cells -as compared to those in NIH3T3 cells. Also, NIH3T3 TrkC cells significantly reduced the levels of PAI-1, TMEPAI-1, and IL-11, but not P15 and P21 relative to the control cells. In addition, these levels were further downregulated in NIH3T3 TrkC-EWSR1-FLI1 cells compared to that in NIH3T3 TrkC or EWSR1-FLI1 cells (Fig. 6B). Although the basal expressions of the anti-apoptotic proteins BCL2L1, BIRC3, BIRC7, TNFAIP3, and IL-1A significantly increased in response to TGF-β1 treatment in NIH3T3 TrkC and EWSR1-FLI1 cells, these levels were upregulated in NIH3T3 TrkC-EWSR1-FLI1 cells (Fig. 6C).

To further understand the correlation between EWSR1-FLI1 and TrkC in the metastatic potential of ES, we tested whether TrkC affects the metastatic ability of EWSR1-FLI1 using the migration assay. Both NIH3T3 TrkC and EWSR1-FLI1 cells had a 2.3-fold increase, while the NIH3T3 TrkC/EWSR1-FLI1 cells had a 4.5-fold increase in the number of migrated cell populations relative to those in the NIH3T3 control cells (Fig. 6D). Correspondingly, the ability of colony formation of NIH3T3 TrkC and EWSR1-FLI1 cells was increased 3.9- and 3.5-fold, respectively, while that of the NIH3T3 TrkC-EWSR1-FLI1 cells was increased 7.8-fold relative to the NIH3T3 control cells (Fig. 6E). We also conducted sphere formation and wound-healing assays to test for another hallmark of cancer cells. TrkC/EWSR1-FLI1 expression significantly increased sphere-forming ability and cell migration relative to the control (Figs. 6F, S11). These results demonstrated that NIH3T3 TrkC and EWSR1-FLI1 cells exhibit a transformed phenotype relative to the control cells. Moreover, the introduction of both TrkC and EWSR1-FLI1 was observed to be more effective in cellular morphological transformation. Consistent with these results, TrkC/EWSR1-FLI1 expression markedly increased cell growth in anchorage-dependent or -independent conditions relative to the NIH3T3 TrkC or EWSR1-FLI1 cells (Fig. 6G). Our observations indicated that TrkC might inhibit TGF-β signaling in ES by regulating EWSR1-FLI1.

### TrkC enhanced stabilization of EWSR1-FLI1

**Fig. 7:**
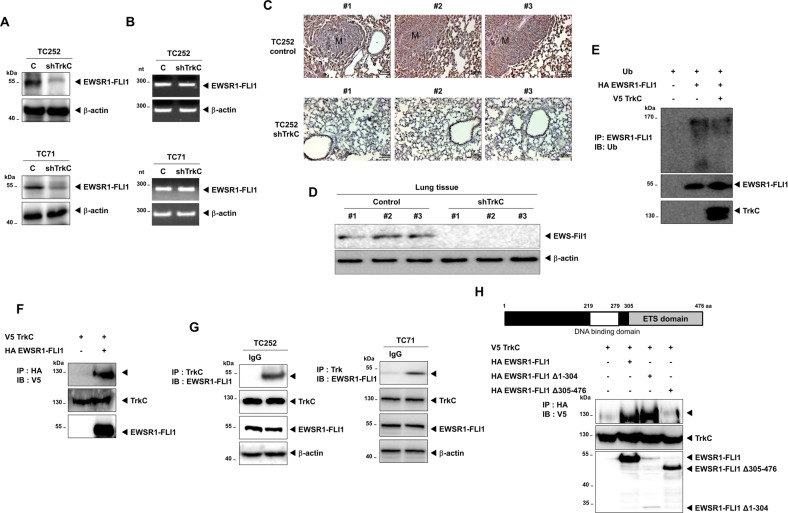
TrkC stabilized EWSR1-FLI1 by inhibiting its proteasomal degradation. **A** Immunoblot analysis of EWSR1-FLI1 in TC252 and TC71 control-shRNA or TrkC-shRNA cells. β-actin was used as a loading control. **B** RT-PCR analysis of *EWSR1-FLI1* in TC252 and TC71 control-shRNA or TrkC-shRNA cells. β-actin was used as a loading control. **C** Representative immunohistochemical images presenting the expression of EWSR1-FLI1 in the lungs excised from individual mice expressing either TC252 control-shRNA or TrkC-shRNA. M, metastatic nodule. **D** Immunoblot analysis of EWSR1-FLI1 in tumor cells recovered from the lungs of individual mice expressing either TC252 control-shRNA or TrkC-shRNA. β-actin was used as a loading control. *n* = 3. **E** Immunoblot analysis of whole-cell lysates and immunoprecipitates derived from 293T cells transfected with the ubiquitin, HA-EWSR1-FLI1, and V5-TrkC constructs, as indicated. **F** Identification of the complex formation of TrkC/EWSR1-FLI1. Immunoblot analysis of whole-cell lysates and immunoprecipitates derived from 293T cells transfected with the V5-TrkC and HA-EWSR1-FLI1 constructs, as indicated. **G** Identification of an endogenous TrkC-EWSR1-FLI1 complex in TC252 cells. Cell lysates were subjected to immunoprecipitation using an anti-IgG or anti-TrkC antibody, followed by immunoblotting with anti-FLI1 and anti-TrkC antibodies. β-actin was used as a loading control. **H** Identification of the region of EWSR1-FLI1 responsible for its interaction with TrkC. Immunoblot analysis of whole-cell lysates and immunoprecipitates derived from 293T cells transfected with the V5-TrkC and HA-EWSR1-FLI1 deletion constructs.

Our above observations suggested that TrkC induces the expression of EWSR1-FLI1 targets, EGR2 or NKX2-2. Therefore, it may be possible that EWSR1-FLI1 functionally links with TrkC to mediate the progression of ES. To characterize the mechanism by which TrkC induces and maintains EWSR1-FLI1-mediated tumor pathogenesis in ES, first, we compared the expression of EWSR1-FLI1 and TrkC between TC252 and TC71 cells. Interestingly, TC252 cells showed an increased TrkC and EWSR1-FLI1 expression relative to TC71 cells (Fig. S12). Second, we examined the expression of EWSR1-FLI1 in TC252 and TC71 control- and TrkC-shRNA cells by RT-PCR and immunoblotting. It was observed that the upregulation of TrkC significantly induced the protein level of EWSR1-FLI1 (Figs. 7A, S13, S14), but it did not affect its mRNA level (Fig. 7B). In addition, we assessed the change in its expression in the lungs of mice injected with either control-shRNA or TrkC-shRNA-expressing TC252 cells. A few metastatic nodules in the lungs of mice carrying TC252 control-shRNA cells retained EWSR1-FLI1 expression at a level similar to that in the control mice. In contrast, its expression was significantly reduced in the lungs of mice with TC252 TrkC-shRNA cells as compared to that in the control mice (Fig. 7C, D). These results indicated that TrkC might be upregulated through the stabilization of EWSR1-FLI1, which is known to be polyubiquitinated and degraded by the proteasome system [11].

We further assessed whether EWSR1-FLI1 stabilization by TrkC was mediated by suppressing EWSR1-FLI1 ubiquitination via the complex formation of TrkC/EWSR1-FLI1. We found that the level of EWSR1-FLI1 ubiquitination was significantly reduced in the presence of TrkC (Fig. 7E), and this reduction was mediated by its direct interaction with TrkC/EWSR1-FLI1 (Figs. 7F, S15). In addition, endogenous TrkC was strongly associated with EWSR1-FLI1 in TC252 and TC71 cells (Fig. 7G). These findings led us to examine whether the tyrosine kinase activity of TrkC is essential for the upregulation of the EWSR1-FLI1. K252a treatment markedly reduced EWSR1-FLI1 expression in TC252 and TC71 cells (Fig. S16), indicating that the upregulation of tyrosine kinase activity by TrkC requires the maintenance of EWS-FLI expression.

Moreover, we identified the protein domain responsible for the interaction ofEWSR1-FLI1 with TrkC. We found The C-terminal region of EWSR1-FLI1 containing the E26 transformation specific (ETS) domain of FLI1 was required for its interaction with TrkC (Fig. 7H). To summarize, the data demonstrated that TrkC enhances the metastatic potential of ES by inhibiting the proteasomal degradation of EWSR1-FLI1 via the formation of a TrkC/EWSR1-FLI1 complex.

## Discussion

Recent studies have demonstrated that upregulation and activation of TrkC have been observed in various types of cancer, such as medullary thyroid, breast, colon, lung, liver, and prostate cancer [15, 16]. These reports suggest that the expression of TrkC may be implicated in the initiation, progression, and metastasis of ES [45, 46]. However, the pathogenesis or progression of ES mediated by TrkC remains unclear due to a lack of knowledge regarding the signaling mechanisms and expression patterns of TrkC in ES. In this study, we observed that TrkC is significantly correlated with the progression and tumorigenicity of ES. TrkC is overexpressed in human ES cell lines and upregulates the expression of molecular signatures (NKX2-2, EGR2, and ADO) associated with human ES’s aggressiveness.

We further explored the effects of TrkC on the tumorigenesis and metastatic potential of ES cells. Interestingly, we found that the tyrosine kinase activity of TrkC mediates the survival, metastatic potential, and acquisition of CSC traits of ES cells through cellular apoptosis blockage. Also, TrkC-mediated apoptosis resistance has been linked to the induction of PI3K/AKT activation and cyclin D1 expression. In our study, TrkC was observed to promote primary Ewing tumor formation and metastasis in vivo, which corroborated the findings of previous reports that early progenitor cells and CSCs are enriched in tumor spheroids [28, 29].

The upregulation of the PI3K/AKT pathway renders Ewing tumors resistant to therapy [47, 48], and *SOX2*, a target of EWSR1-FLI1, inhibits ES cell apoptosis by activating the PI3K/AKT pathway [49]. These results, combined with our study’s findings, suggest that TrkC expression may be correlated with EWSR1-FLI1 in ES. However, the underlying mechanism by which TrkC regulates the progression of ES remains to be elucidated. TGF-β signaling induces growth arrest or cell death [50] and inhibits the cell plasticity of ES by activating Smad2 and Smad3 [39].

In the present study, we identified a new molecular and functional network incorporating TrkC in the pathogenesis of ES. TrkC regulates EWS-FLI-mediated proliferation and metastatic ES ability by suppressing TGF-β signaling. Although our previous report proposes that TrkC forms a complex with TGFBR2, thereby inhibiting its activation [40], it has not been reported whether TrkC inhibits TGF-β signaling by suppressing mRNA expression of TGFBR2 by stabilizing EWSR1-FLI1 to promote induced ES cell survival and metastatic dissemination.

Overall, this study, for the first time, identified TrkC as a master regulator of ES, and it is likely a potential therapeutic target for ES. Previous studies have demonstrated that EWSR1-FLI1 and microRNA-20b reduce *TGFBR2* expression by interacting with its promoter [43, 44, 51]. Remarkably, we found that TrkC stabilized the EWSR1-FLI1 protein by inhibiting its proteasomal degradation, thus reducing *TGFBR2* expression. Together, these results indicate that TrkC promotes the survival, tumorigenesis, and metastasis of ES cells by preventing the degradation of EWSR1-FLI1 and facilitating the activation of its target genes. An implication of this is the possibility that apart from TrkC, other molecular components of the TrkC-associated pathway may be involved in the pathogenesis of Ewing tumors. To further understand the role of TrkC in the development of novel anticancer therapeutics, its involvement in the tumorigenicity and metastasis of ES needs to be better understood.

## Materials and methods

### Cell culture and reagents

Human ES tumor cell lines (A4753, STA-ETA, VH67, WE68, and CADO-ES1) were obtained as a generous gift from M. C. Manara (SSN Emilia Romagna Istituti Ortopedici Rizzoli IRCCS, Bologna, Italy), and RE-DS and TC71 were kindly provided by H. Kovar (Children’s Cancer Research Institute, Vienna, Austria). Other breast and liver cancer cell lines obtained from American Type Culture Collection (Manassas, VA, USA) and maintained as described previously [16, 52]. ES Cells were maintained in RPMI 1640 (Gibco, Grand Island, NY) containing 10% FBS. The protein kinase inhibitor K252a was purchased from Calbiochem (Gibbstown, NJ).

### TrkC-shRNA screening

Human TrkC knockdown was performed using MISSION shRNA pLKO.1-puro plasmids as previously described [18, 53]. TC252 and TC71 ES cells were infected with the TrkC-shRNA lentivirus and selected with 1 mg/ml puromycin.

### Anchorage-independent cell growth, transfection, a reporter assay, viral production, antibodies, apoptosis, immunoblotting, and immunoprecipitation analysis

All analyses were performed as previously described [18, 53]. The following antibodies were used in this study: anti-phospho-Smad2, anti- phospho-Smad3, anti-Smad2, anti-Smad3, anti-cyclin D1, and anti-phospho-AKT (Cell Signaling Technology, Danvers, MA); anti-Fli1 (Abcam, Cambridge, MA); anti-HA (Santa Cruz Biotechnology, Dallas, TX); anti-V5 (Life Technologies, Grand Island, NY); anti-β-actin (Sigma-Aldrich, St. Louis, MO).

### RNA preparation, RT-PCR, and quantitative RT-PCR analysis

The primer sequences are listed in the supplemental experimental procedures in Table S1. RNA Preparation, RT-PCR, and Quantitative RT-PCR Analysis were performed as previously described [54]. Specific human TrkC (Hs00176797_m1) and human 18S (Hs99999901_s1) quantitative probes for Taqman RT-PCR were obtained from Applied Biosystems. The expression of TGFBR2 was quantified by SYBR green (Applied Biosystems).

### Immunohistochemistry

The tissues of mice injected with TC252 TrkC-shRNA cells were subjected to immunohistochemistry as previously described [55].

### In silico analysis of clinical microarray data

TrkC, NK2 homeobox 2 (NKX2-2), Early growth response protein 2 (EGR2), cysteamine dioxygenase (ADO), and Tat-activating regulatory DNA-binding protein (TARDBP) expression signatures in the datasets obtained from patients diagnosed with ES were extracted and subjected to the In silico analysis of the published clinical microarray using the GSE12102 [19] and GSE34620 [21]. The boxplot graphs were plotted with gene expression using GraphPad Prism v 5.0 (GraphPad Software, Inc.), and *P* < 0.05 was considered statistically significant.

### Animal studies

Animal studies were performed as previously described [54]. For tumorigenicity studies, 1 × 10^6^ cells suspended in 50 μl phosphate buffer saline /Matrigel (BD Biosciences) were subcutaneously injected into the left and right hind flank regions of anesthetized mice (5 weeks old, *n* = 7). Mice were euthanized at 6 weeks post-injection, and primary tumors were excised for analysis.

For extravasation studies, TC252 control-shRNA or TrkC-shRNA cells (1 × 10^6^ cells) were injected into the tail vein of female BALB/c mice (5 weeks old, *n* = 7). After 42 days, the lungs were excised, fixed in 10% formalin, paraffin-embedded, and sectioned for analysis.

Mice handling was performed in compliance with protocols approved by the Institutional Animal Care and Use Committee (IACUC) of Gachon University (Approval No. LCDI-2015-0071).

### Statistical analysis

Data are expressed as mean ± standard error of the mean (SEM). Statistical analyses of the data were conducted via Student’s *t* test (two-tailed) and ANOVA. Differences were considered statistically significant at *P* < 0.05 or *P* < 0.001.

## Supplementary information

Reproducibility checklist

Supplementary Figure legends

Supplementary Table 1

Supplementary Figure 1

Supplementary Figure 2

Supplementary Figure 3

Supplementary Figure 4

Supplementary Figure 5

Supplementary Figure 6

Supplementary Figure 7

Supplementary Figure 8

Supplementary Figure 9

Supplementary Figure 10

Supplementary Figure 11

Supplementary Figure 12

Supplementary Figure 13

Supplementary Figure 14

Supplementary Figure 15

Supplementary Figure 16

Original western blots

## Data Availability

All data generated and analyzed during this study are included in this published article and its supplementary information file.
