# Supplementary material for: TrkC, a novel prognostic marker, induces and maintains cell survival and metastatic dissemination of Ewing sarcoma by inhibiting EWSR1-FLI1 degradation
Source: Cell Death Dis. 2022 Sep 28;13(9):836. doi: 10.1038/s41419-022-05275-w (PMC9519565; doi:10.1038/s41419-022-05275-w)
Supplement: Supplementary file 2 — Supplementary Figure legends [file 41419_2022_5275_MOESM2_ESM.docx]

**Supplementary Figure legends**

**Figure S1. TrkC expression remained unaltered between primary ES and relapsed ES.** (A) Box-and-whisker (Tukey) plots are shown for the expression of TrkC in patients with primary and relapsed ES. The TrkC level was extracted from the Scotlandi microarray dataset (GSE12102) and averaged for each tumor. *P* values; NS (not significant), *t*-test.

**Figure S2. Upregulation of TrkC induced cell migration.** The graph shows the cell covered rate (%) of TC252 and TC71 ES cells at 24h post-wound generation; quantification performed using the ImageJ software. n = 3. *P* values < 0.05 (**); *t*-test.

**Figure S3. TARDBP expression remained unaltered for high and low TrkC expressing cells.** Box-and-whisker (Tukey) plots are shown for the expression of TARDBP in patients with ES (n=117); data obtained from the Postel-Vinay dataset (GSE34620) after being divided into high and low TrkC expressers. *P* values; NS (not significant), *t*-test.

**Figure S4. Transfection of TrkC-shRNA reduced *TrkC* Expression.** RT-PCR analysis of *TrkC* expression in TC252 and TC71 cells control-shRNA or TrkC-shRNA cells. GAPDH was used as a loading control. The densitometric intensity of TrkC expression in these cells was quantified using the ImageJ software. n = 3. *P* values < 0.05 (**); *t*-test.

**Figure S5. Downregulation of TrkC reduced cell migration of ES cells.** The graph shows the cell covered rate (%) of TC252, TC71, and its TrkC-shRNA cells at 24 h post-wound generation; quantification was performed using the ImageJ software. n = 3. *P* values < 0.05 (**); *t*-test.

**Figure S6. The tyrosine kinase activity of TrkC was required for the cell growth and survival of ES cells.** (A) Bright-phase microscopy images of the formation of spheroid colonies and quantification of cell growth of TC252 and TC71 control-shRNA or TrkC-shRNA cells in anchorage-independent conditions. Each data point represents the mean of cells counted in three dishes. **TC252 or TC71 control cells versus respective TC252-TrkC shRNA cells, *P* values < 0.05; *t*-test. (B) Quantification of cell growth of TC252 and TC71 cells treated with 25 or 50 nM K252a. Each data point represents the mean of cells counted in three dishes. **TC252 or TC71 control cells versus respective cells treated with 25 or 50nM K252a, *P* values < 0.05; *t*-test. (C) Bright-phase microscopy images of the formation of spheroid colonies and quantification of cell growth of TC252 and TC71 cells in anchorage-independent conditions. Each data point represents the mean of cells counted in three dishes. TC252 or TC71 control cells versus respective cells treated with 25 or 50nM K252a, *P* values < 0.05 (**); *t*-test.

**Figure S7. Upregulation of TrkC induced the expression of phospho-AKT and cyclin D1.** The ImageJ software quantified the densitometric intensity of protein bands of phospho-AKT and cyclin D1 in TC252 control-shRNA and TrkC-shRNA cells. n = 3. *P* values < 0.001 (***); One-way ANOVA.

**Figure S8. The role of TrkC in TGF-β-mediated tumor suppressor activity.** The activity of TGF-β1-responsive 3TP luciferase reporter in TC252 and TC71 control-shRNA or TrkC-shRNA cells. Luciferase activity was measured at 24 h after TGF-β1 (5 ng/mL) treatment. n = 3. *P* values < 0.05 (**); *t*-test.

**Figure S9. Upregulation of TrkC markedly reduces TGFBR2 expression.** Box-and-whisker (Tukey) plots are shown for the expression of TGFBR2 in patients with ES (n=117) from the Postel-Vinay dataset (GSE34620) after being divided into high and low TrkC expressers. *P* values < 0.05 (**); *t*-test.

**Figure S10. TrkC expression significantly reduced EWSR1-FLI1-mediated suppression of TGF-β signaling.** The activity of TGF-β1-responsive SBE or 3TP luciferase reporter in NIH3T3 cells expressing control, TrkC, EWSR1-FLI1, and TrkC/EWSR1-FLI1. Luciferase activity was measured at 24 h after TGF-β1 (5 ng/mL) treatment. n = 3. *P* values < 0.05 (**); *t*-test.

**Figure S11.** **TrkC expression significantly induced EWSR1-FLI1-mediated** **cell migration.** (A) Wound healing assay of NIH3T3 cells expressing control, TrkC, EWSR1-FLI1, and TrkC/EWSR1-FLI1. Wound closures were photographed at 0 and 24 h after wound generation.

**Figure S12.** **Comparison of EWSR1-FLI1 and TrkC** e**xpression.** Immunoblot analysis of EWSR1-FLI1 and TrkC expression in TC252 and TC71 ES cells. β-actin was used as a loading control.

**Figure S13. Downregulation of TrkC reduced the expression of EWSR1-FLI1** **in ES cells.** The graph shows the relative intensity of protein bands of EWSR1-FLI1 in TC252, TC71, and its TrkC-shRNA cells. The densitometric intensity of EWSR1-FLI1 was quantified using the ImageJ software. n = 3. *P* values < 0.05 (**); *t*-test.

**Figure S14. Upregulation of TrkC expression significantly induced EWSR1-FLI1 expression.** Immunoblot analysis of EWSR1-FLI1 and TrkC expression in TC252 and TC71 cells after transfection with V5-TrkC constructs.

**Figure S15. Interaction of TrkC with EWSR1-FLI1.** Immunoblot analysis of whole-cell lysates and immunoprecipitates derived from 293T cells transfected with the HA-EWSR1-FLI1 and V5-TrkC constructs.

**Figure S16. Suppression of tyrosine kinase activity of TrkC reduced the EWSR1-FLI1 expression.** Immunoblot analysis of EWSR1-FLI1 in TC252 and TC71 ES cells treated with K252a, a Trk tyrosine kinase inhibitor. β-actin was used as a loading control.
