# Supplementary material for: TrkC, a novel prognostic marker, induces and maintains cell survival and metastatic dissemination of Ewing sarcoma by inhibiting EWSR1-FLI1 degradation
Source: Cell Death Dis. 2022 Sep 28;13(9):836. doi: 10.1038/s41419-022-05275-w (PMC9519565; doi:10.1038/s41419-022-05275-w)
Supplement: Supplementary file 3 — Supplementary Table 1 [file 41419_2022_5275_MOESM3_ESM.docx]

**Table S1. Primer sequences for RT-PCR and quantitative RT-PCR**

| **Quantitative RT-PCR Primers** | |
| --- | --- |
| Gene | Primers |
| **Human TGFBR2** | F: 5’-CAATCCTGACTTGTTGCTAGTCATATT-3’  R: 5’-ATGACAGATATGGCAACTCCCA-3’ |
| **Human NKX2.2** | F: 5′-GGGACGCCGGCAAGA-3′  R: 5′-TAGGTCTGCGCCTTGGAGAA-3′ |
| **Human EGR2** | F: 5’- GCCGTAGACAAAATCCCAG-3’  R: 5’- CCACTCCGTTCATCTGGTC-3’ |
| **Human 18S** | F: 5’-ACCGCAGCTAGGAATAATGGA-3’  R: 5’-GCCTCAGTTCCGAAAACCA-3’ |
| **RT-PCR Primers** | |
| Gene | Primers |
| **Human BIRC3** | F: 5’- GTATTCCACTTTTCCTGCTG-3’  R: 5’- TTTTCTGTACCCGGAAGTAA-3’ |
| **Human BCL2L1** | F: 5’- GTGGAAGAGAACAGGACTGA -3’  R: 5’- AAAGCTCTGATATGCTGTCC-3’ |
| **Human TNFAIP3** | F: 5’- GGATCTGCAGTACTTGCTTC-3’  R: 5’- TCTGGAGTCCCAAAATACAC -3’ |
| **Human BIRC7** | F: 5’- GACCTAAAGACAGTGCCAAG-3’  R: 5’- GTCATAGAAGGAGGCCAGA-3’ |
| **Human IL-1A** | F: 5’- TGAAGAAGAGACGGTTGAGT-3’  R: 5’- GCATCATCCTTTGATGACTT-3’ |
| **Human P21** | F: 5’-GGCTTCATGCCAGCTACTTC-3’  R: 5’-CCCTAGGCTGTGCTCACTTC-3’ |
| **Human P15** | F: 5’-CGCCCACAACGACTTTATTT-3’  R: 5’-CACCAGGTCCAGTCAAGGAT-3’ |
| **Human PAI-1** | F: 5’-TCTCAGGAAGTCCAGCCACT-3’  R: 5’-ACCCTCTGGCTGGTAGGTTT-3’ |
| **Human IL-11** | F: 5’- GCAATCTGAGGTCACTGCAA-3’  R: 5’- CAGGGTGACTTGTGGAACCT-3’ |
| **Human TMEPAI-1** | F: 5’-GAGGTGCCTAGCTTGGTGAG-3’  R: 5’-GCAGAGAAGCCGAGAGCTTA-3’ |
| **Human EWS-FLI1** | F: 5’- GCACCTCCATCCTACCCTCCT-3’  R: 5’- TGGCAGTGGGTGGGTCTTCAT-3’ |
| **Human β-actin** | F: 5’-TCACCCACACTGTGCCCATCTACGA-3’  R: 5’-CAGCGGAACCGCTCATTGCCAATGG-3’ |
