# Supplementary figures and images for: TrkC, a novel prognostic marker, induces and maintains cell survival and metastatic dissemination of Ewing sarcoma by inhibiting EWSR1-FLI1 degradation
Source: Cell Death Dis. 2022 Sep 28;13(9):836. doi: 10.1038/s41419-022-05275-w (PMC9519565; doi:10.1038/s41419-022-05275-w)

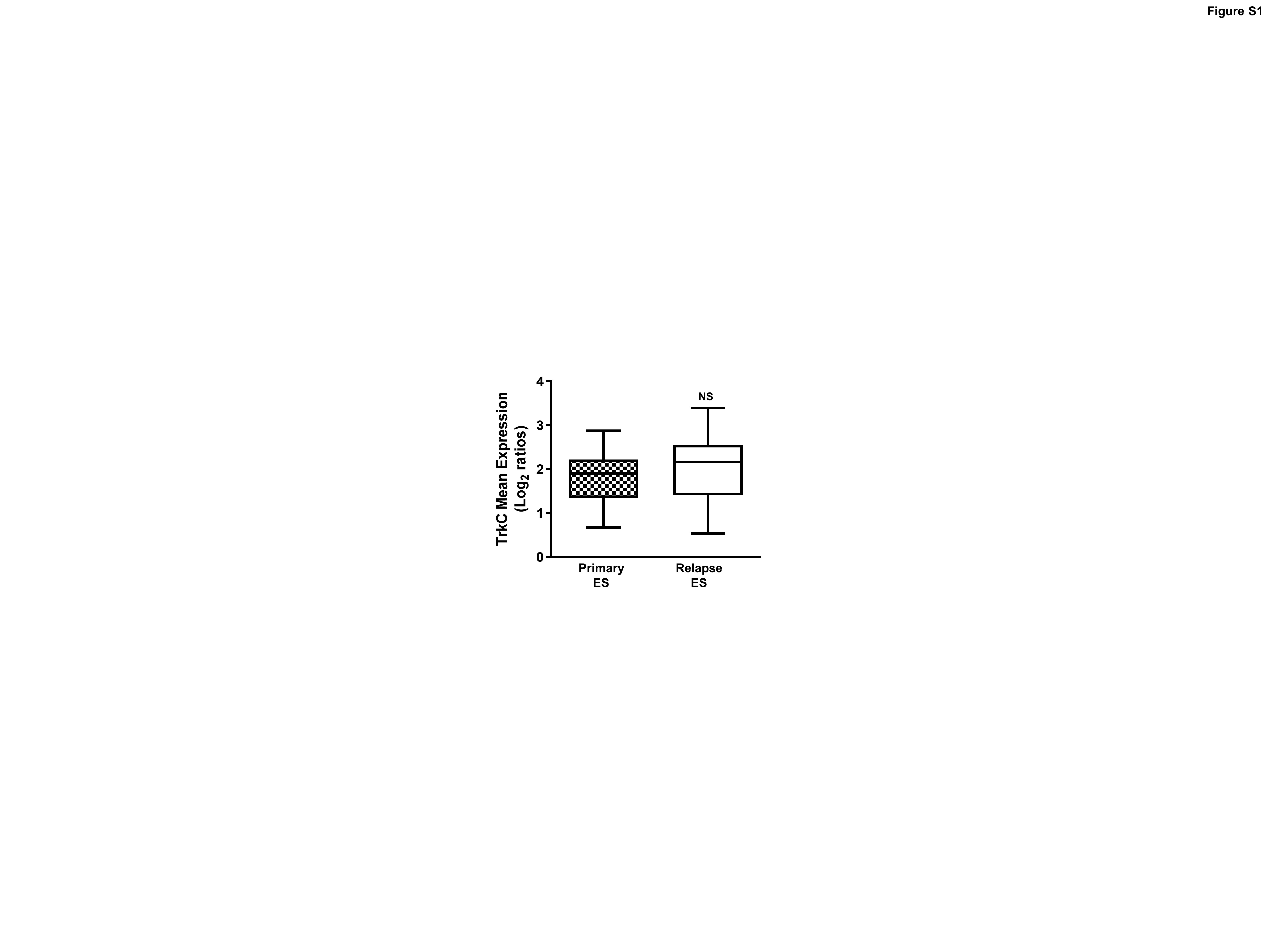

Supplement: Supplementary file 4 — Supplementary Figure 1 [file 41419_2022_5275_MOESM4_ESM.jpg]

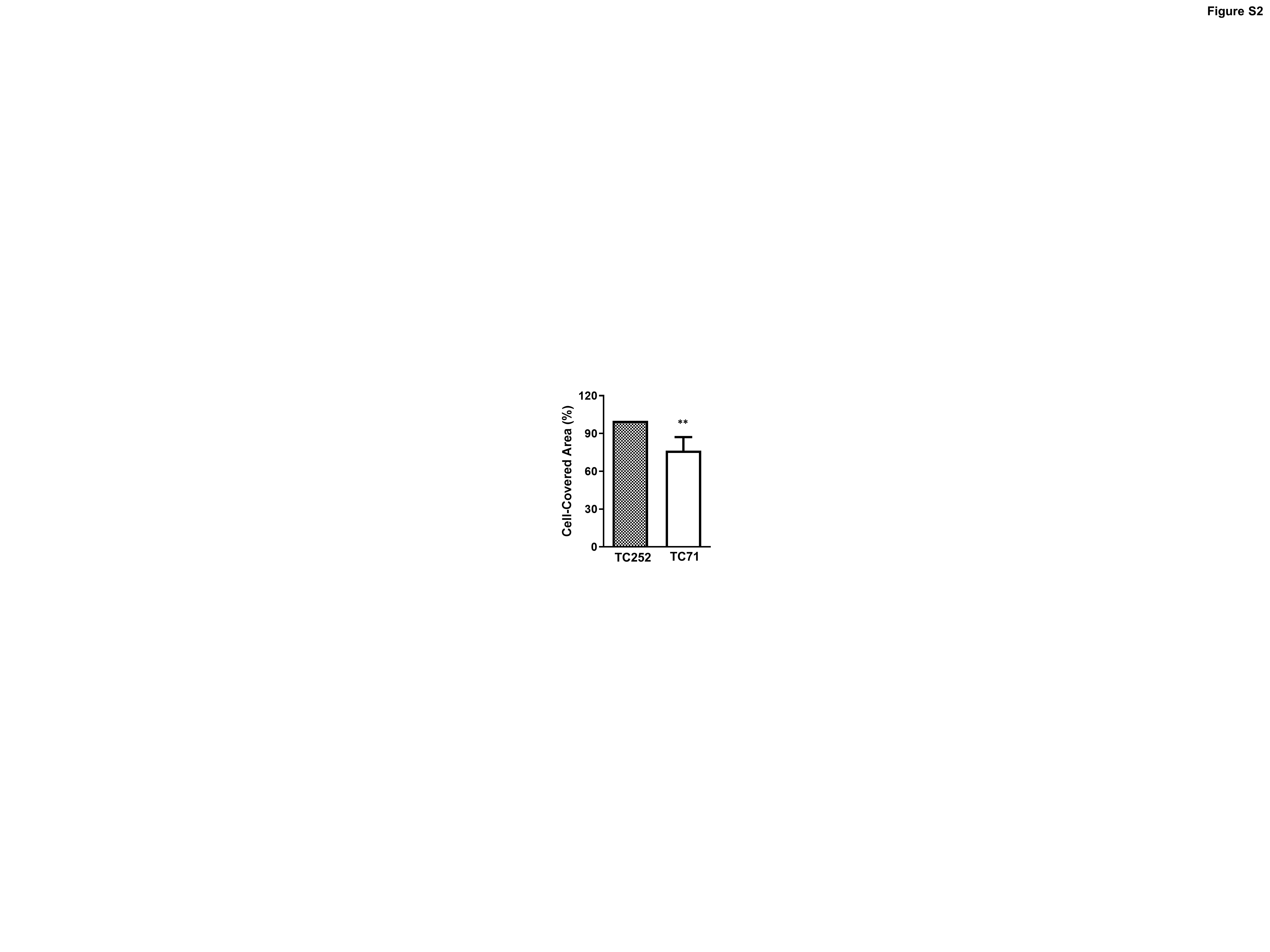

Supplement: Supplementary file 5 — Supplementary Figure 2 [file 41419_2022_5275_MOESM5_ESM.jpg]

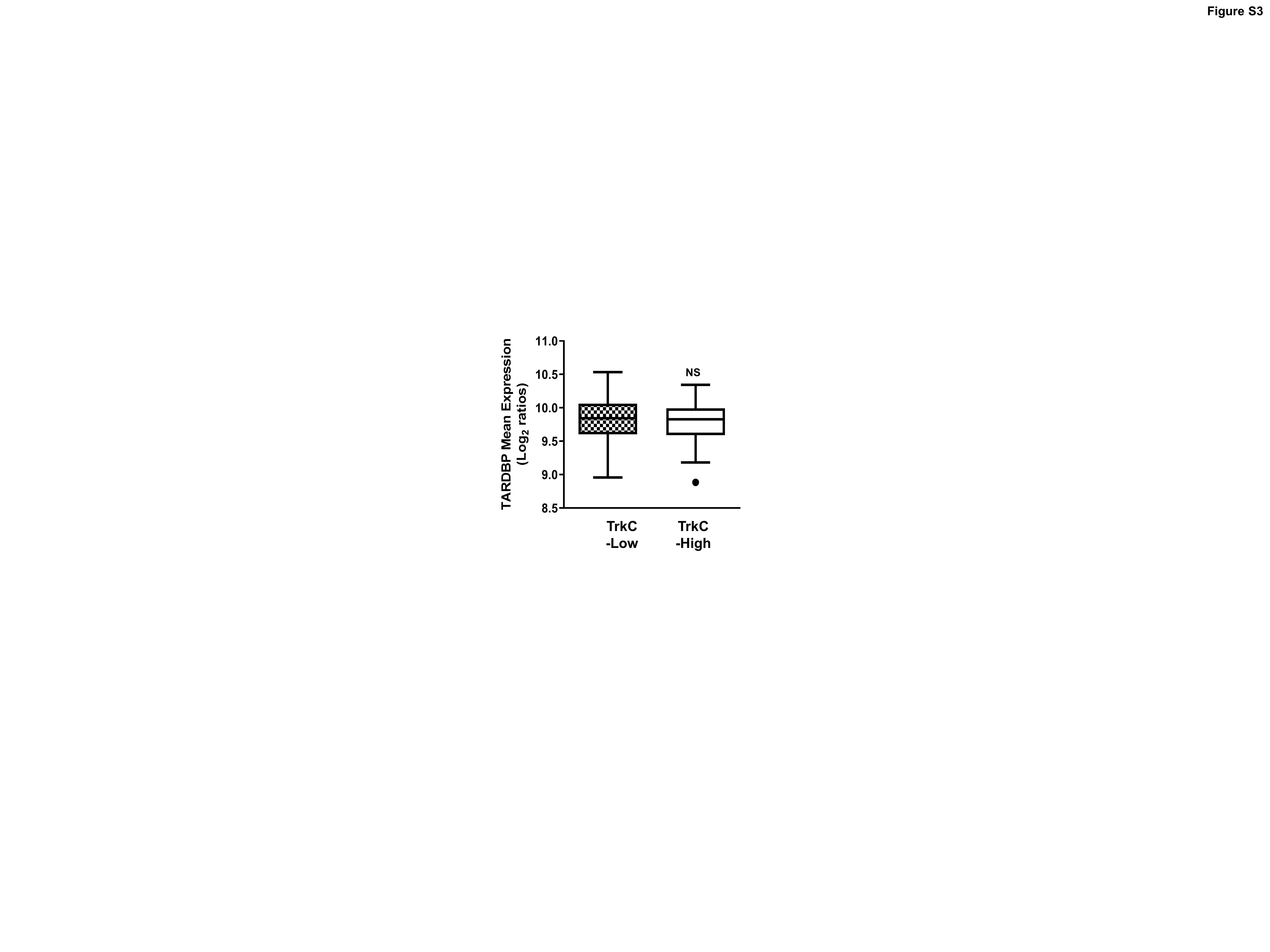

Supplement: Supplementary file 6 — Supplementary Figure 3 [file 41419_2022_5275_MOESM6_ESM.jpg]

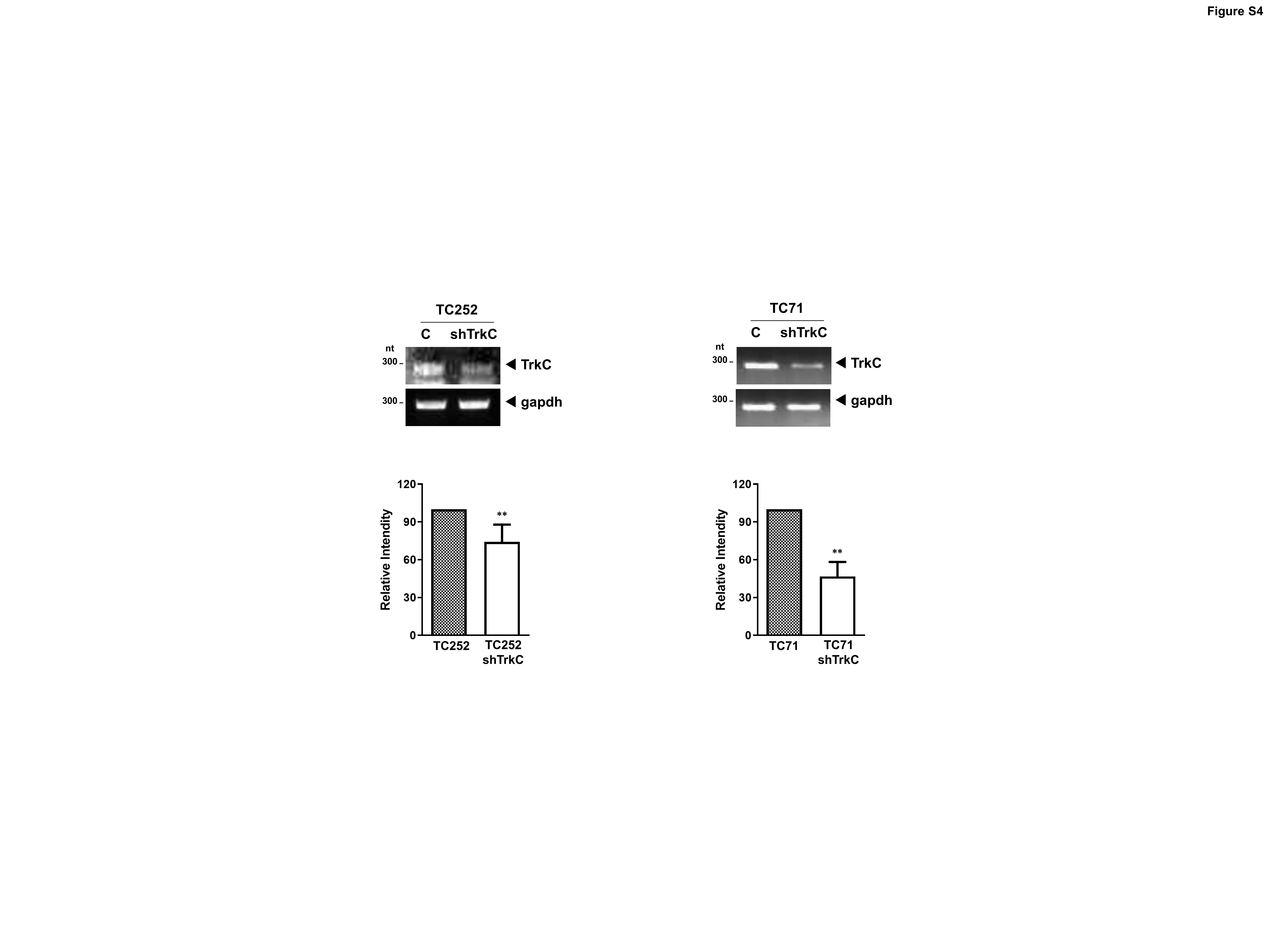

Supplement: Supplementary file 7 — Supplementary Figure 4 [file 41419_2022_5275_MOESM7_ESM.jpg]

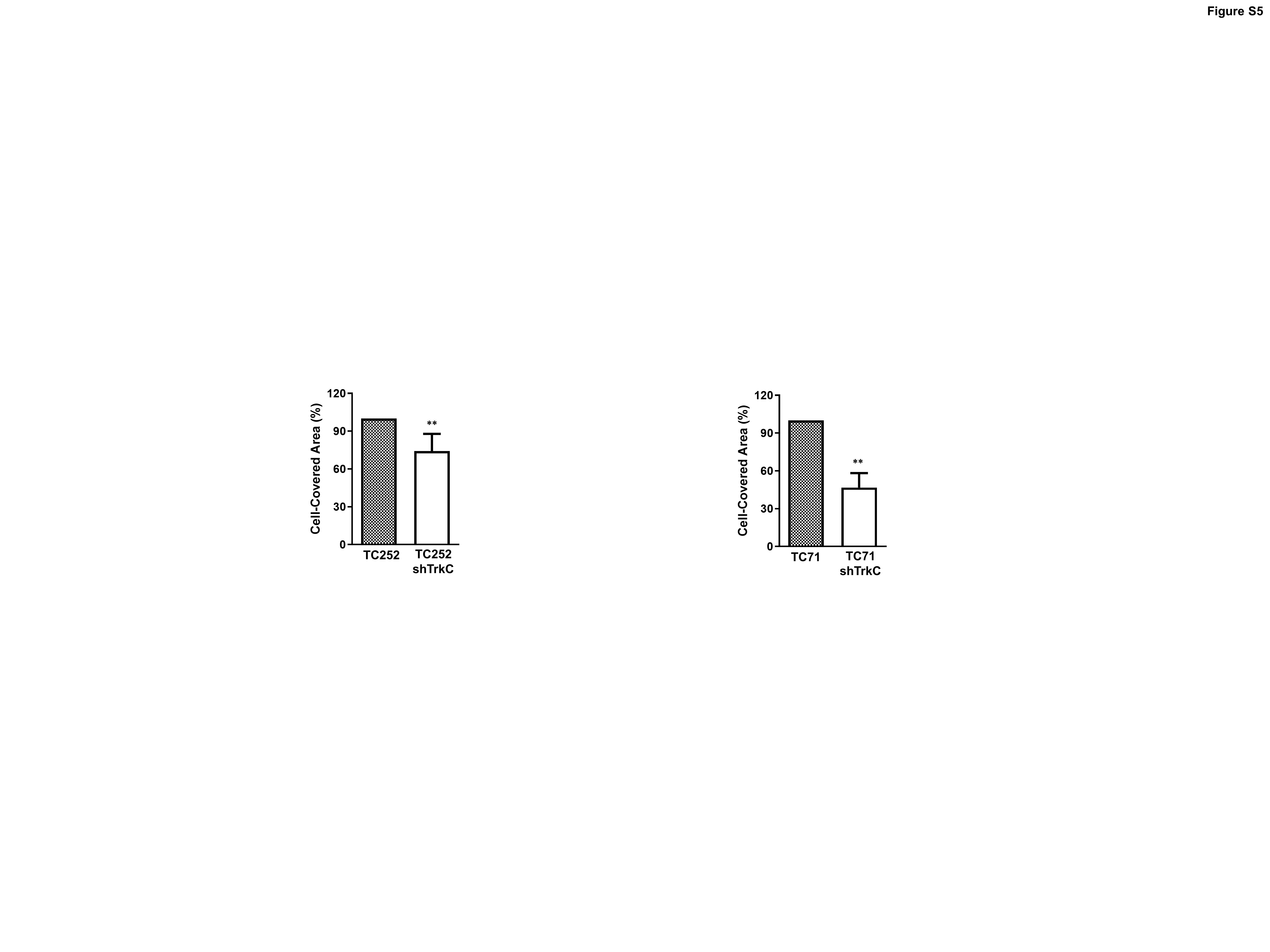

Supplement: Supplementary file 8 — Supplementary Figure 5 [file 41419_2022_5275_MOESM8_ESM.jpg]

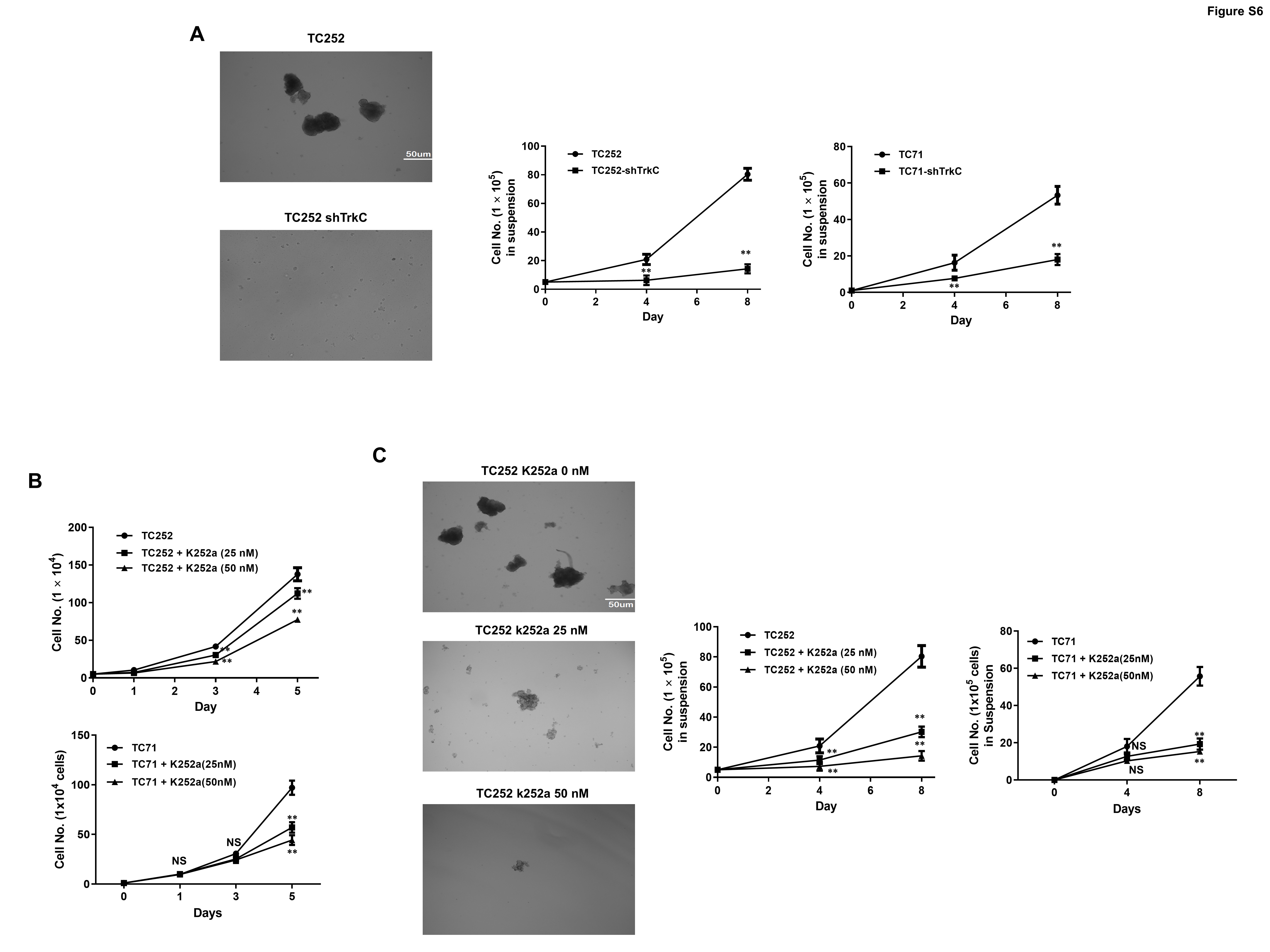

Supplement: Supplementary file 9 — Supplementary Figure 6 [file 41419_2022_5275_MOESM9_ESM.jpg]

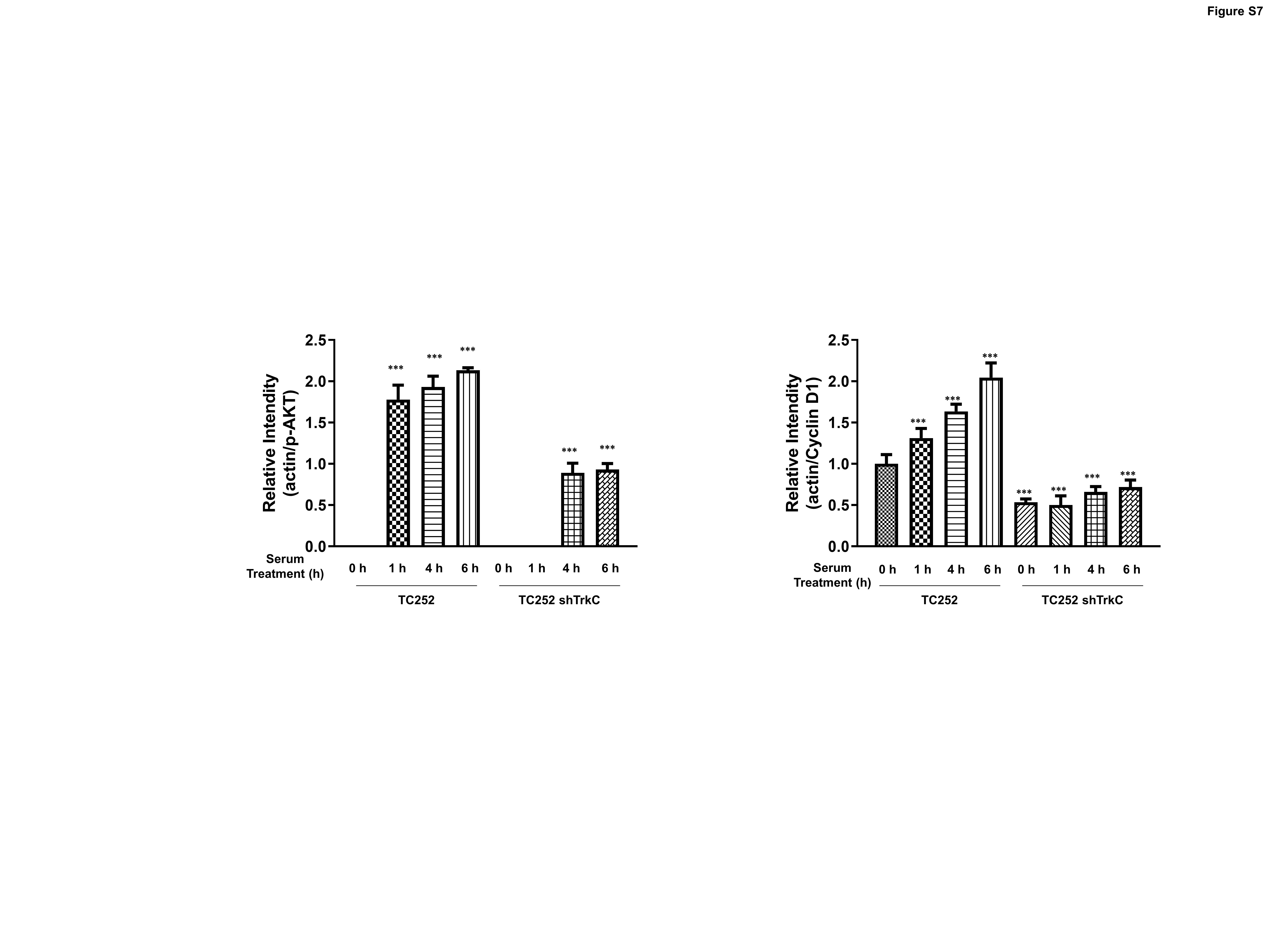

Supplement: Supplementary file 10 — Supplementary Figure 7 [file 41419_2022_5275_MOESM10_ESM.jpg]

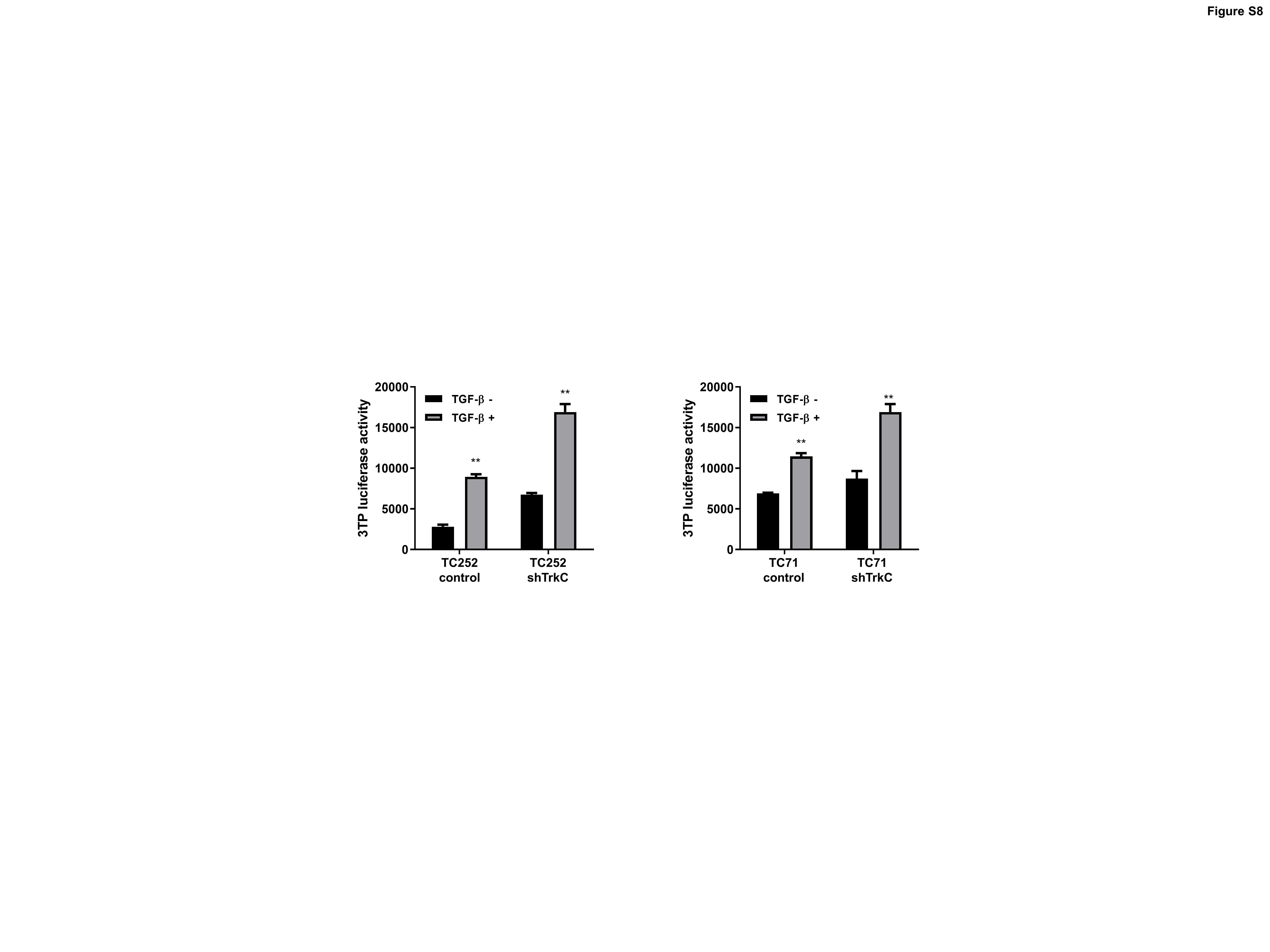

Supplement: Supplementary file 11 — Supplementary Figure 8 [file 41419_2022_5275_MOESM11_ESM.jpg]

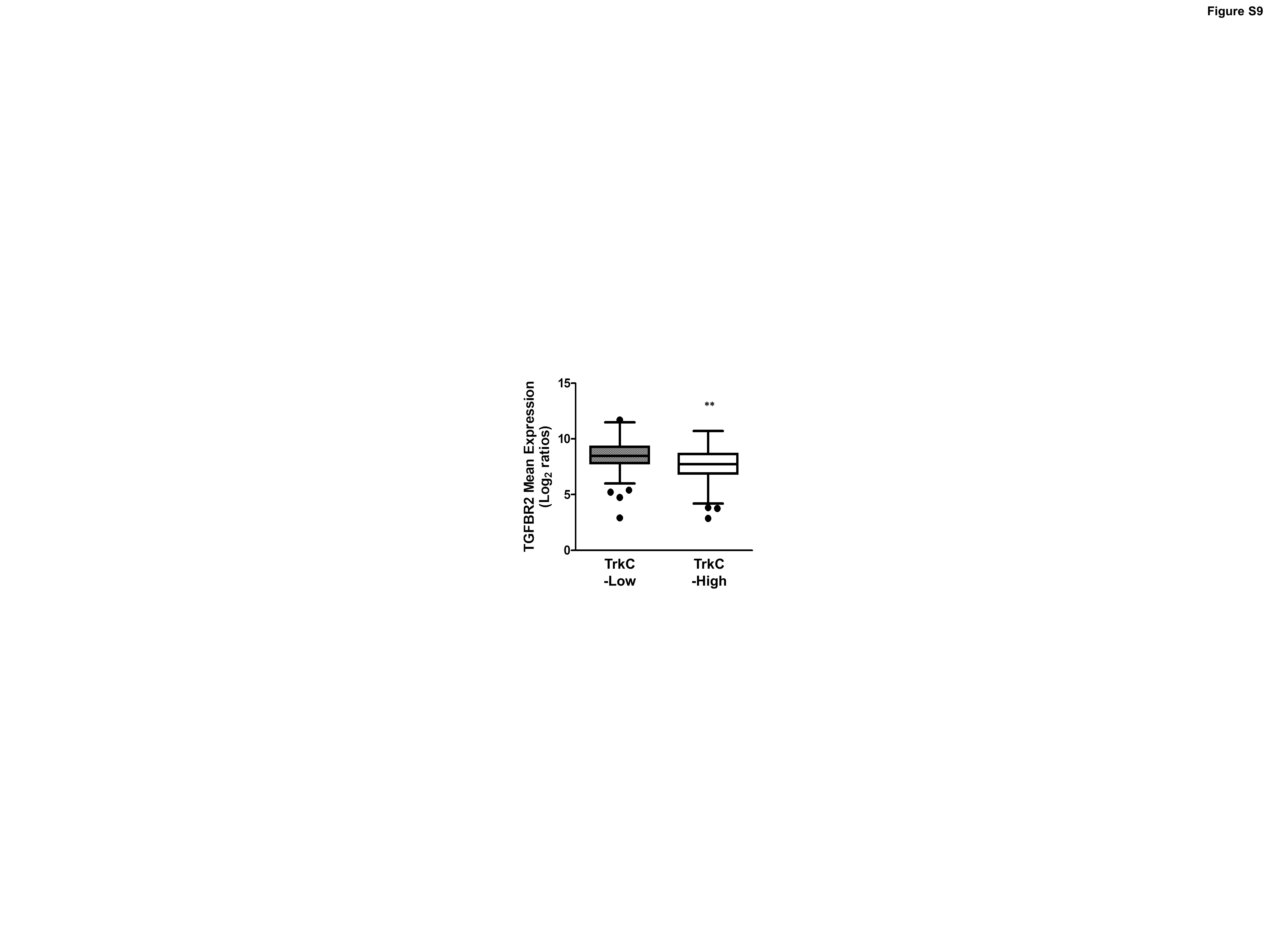

Supplement: Supplementary file 12 — Supplementary Figure 9 [file 41419_2022_5275_MOESM12_ESM.jpg]

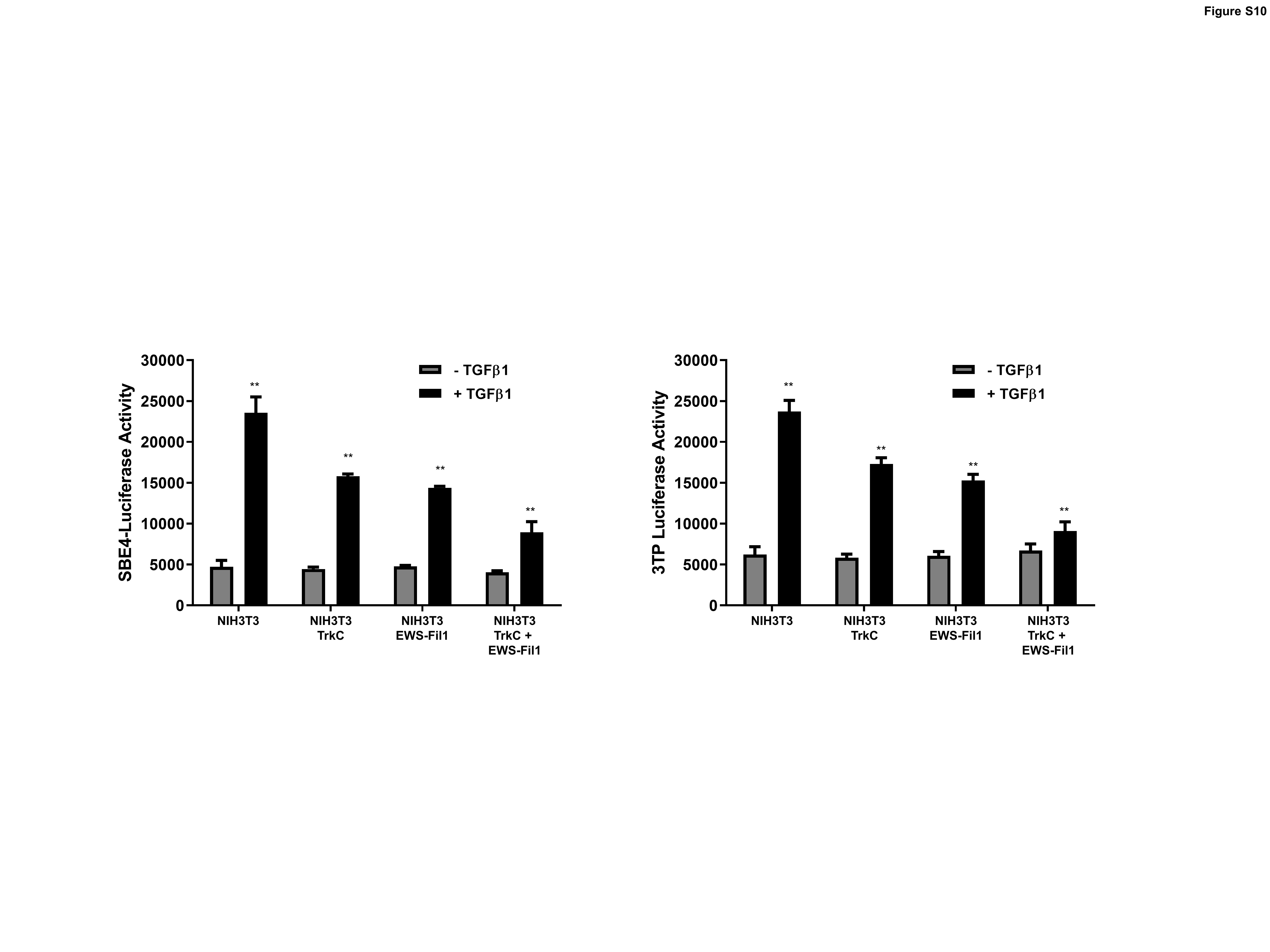

Supplement: Supplementary file 13 — Supplementary Figure 10 [file 41419_2022_5275_MOESM13_ESM.jpg]

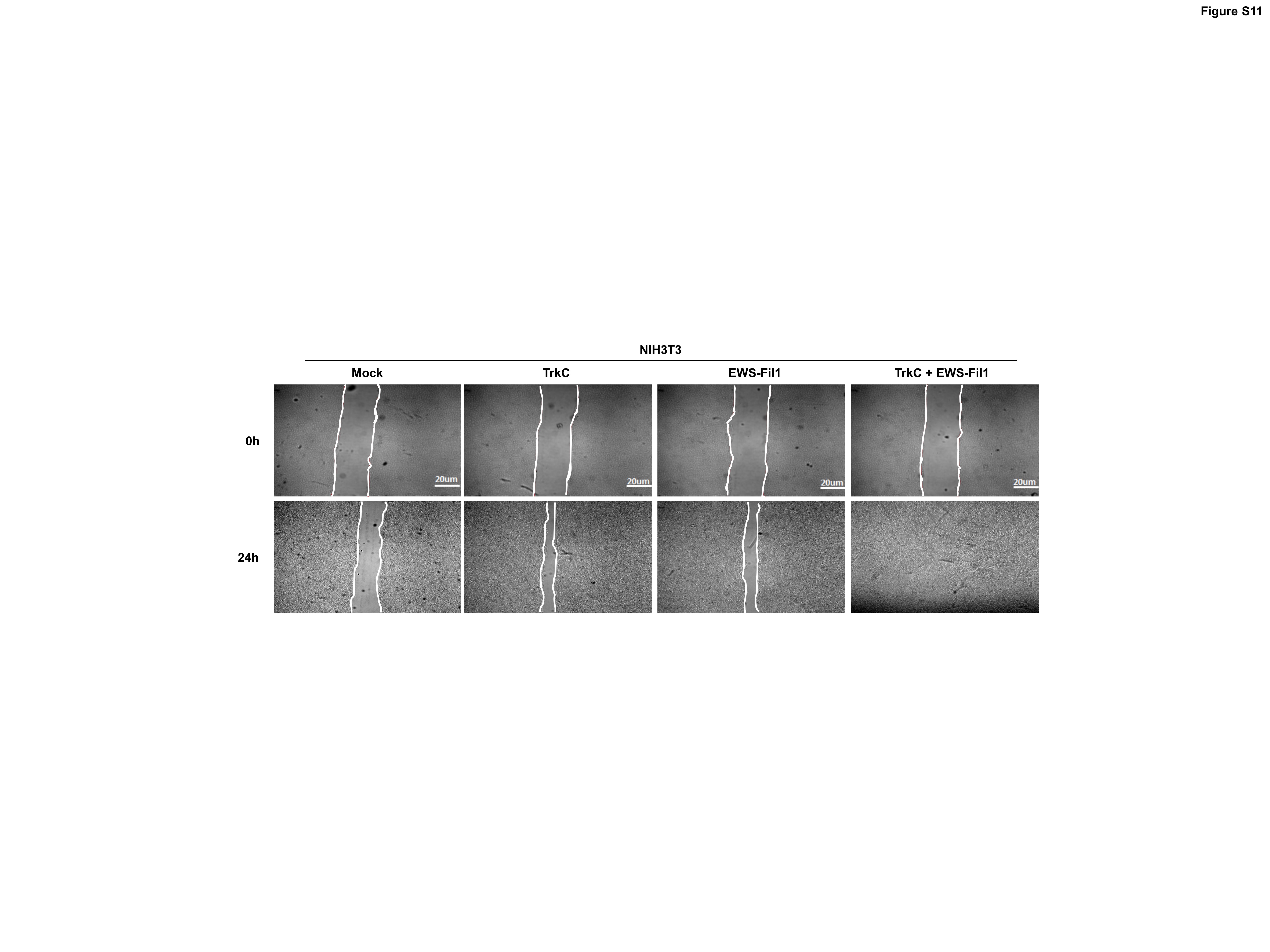

Supplement: Supplementary file 14 — Supplementary Figure 11 [file 41419_2022_5275_MOESM14_ESM.jpg]

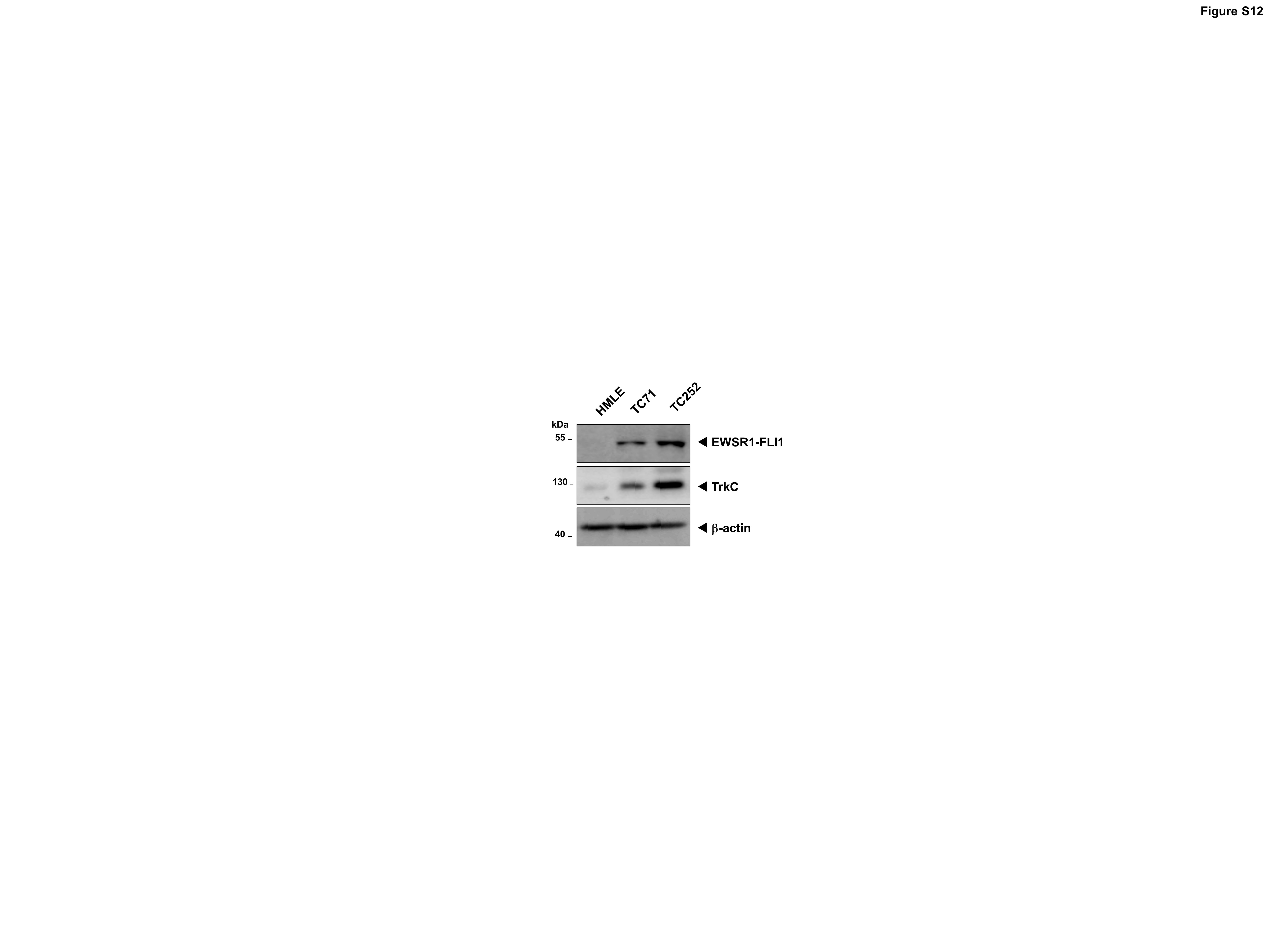

Supplement: Supplementary file 15 — Supplementary Figure 12 [file 41419_2022_5275_MOESM15_ESM.jpg]

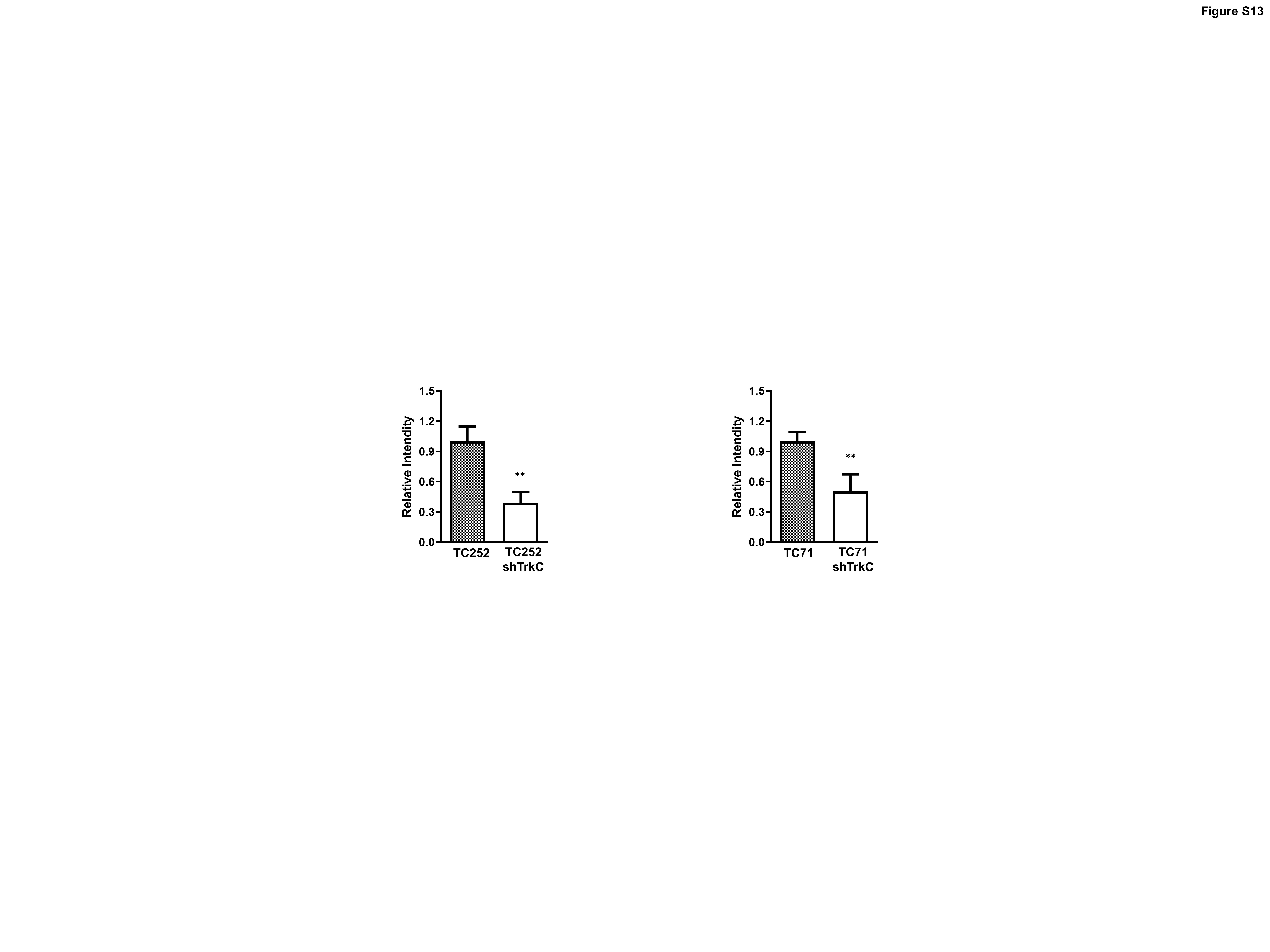

Supplement: Supplementary file 16 — Supplementary Figure 13 [file 41419_2022_5275_MOESM16_ESM.jpg]

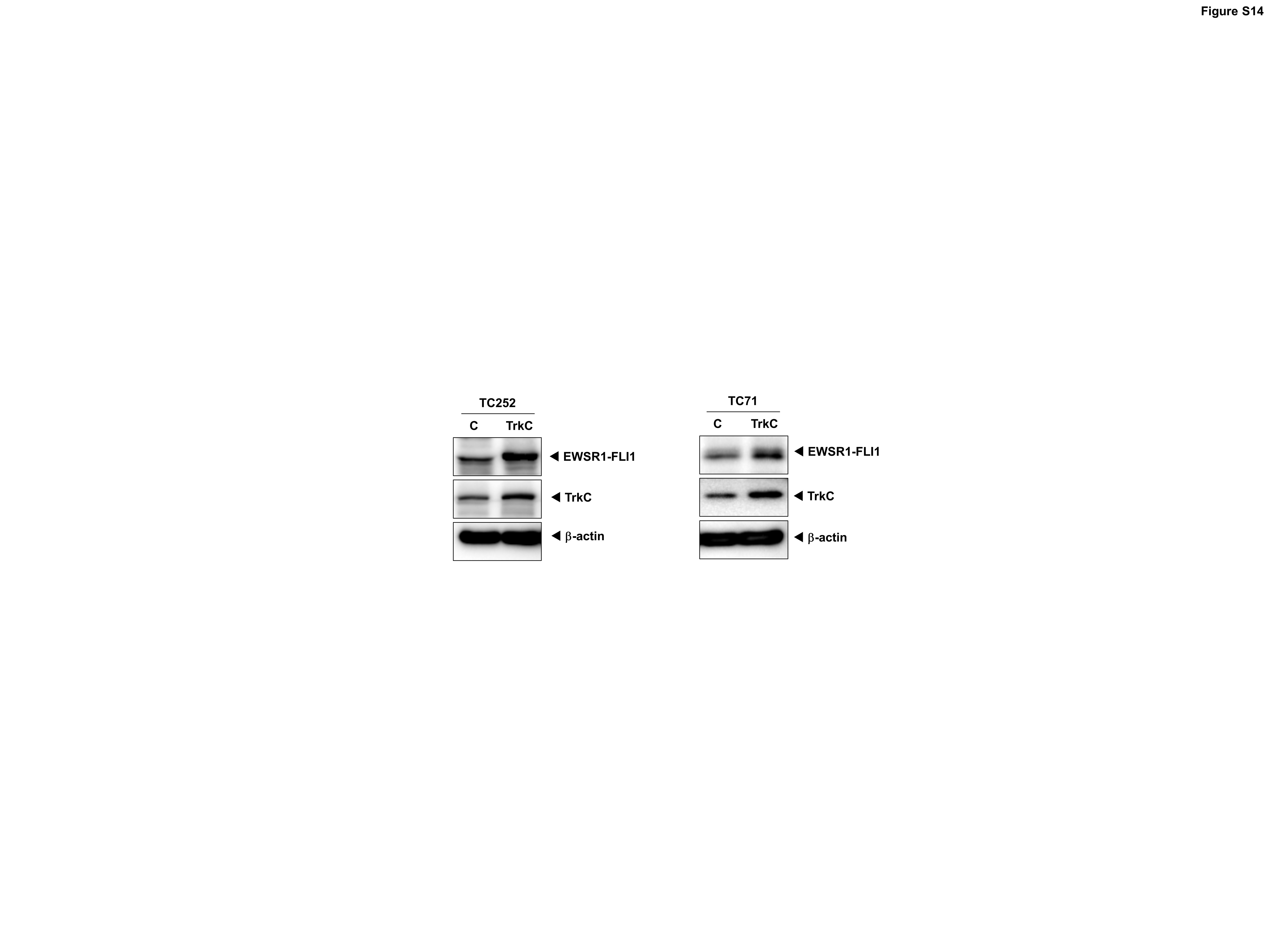

Supplement: Supplementary file 17 — Supplementary Figure 14 [file 41419_2022_5275_MOESM17_ESM.jpg]

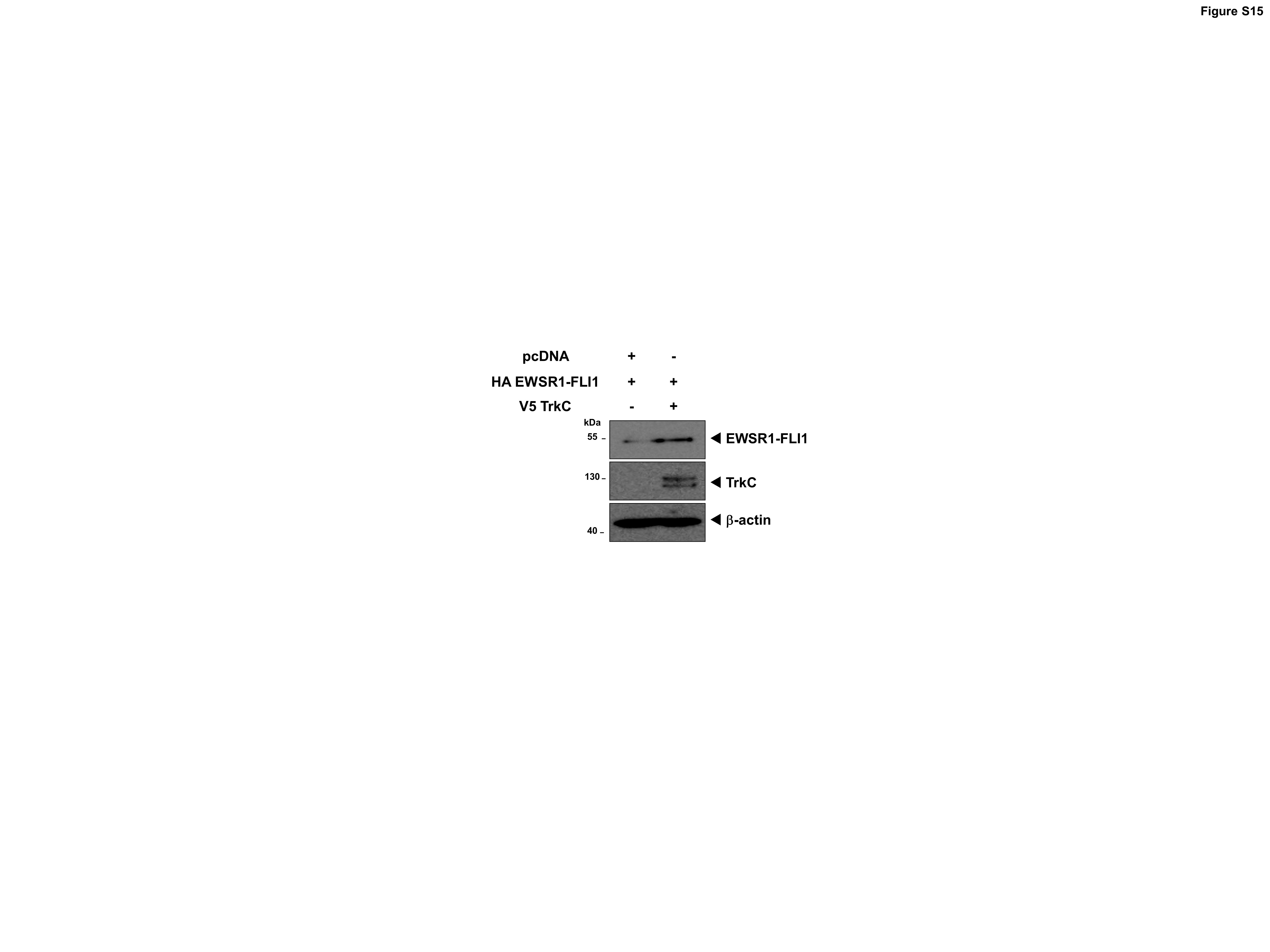

Supplement: Supplementary file 18 — Supplementary Figure 15 [file 41419_2022_5275_MOESM18_ESM.jpg]

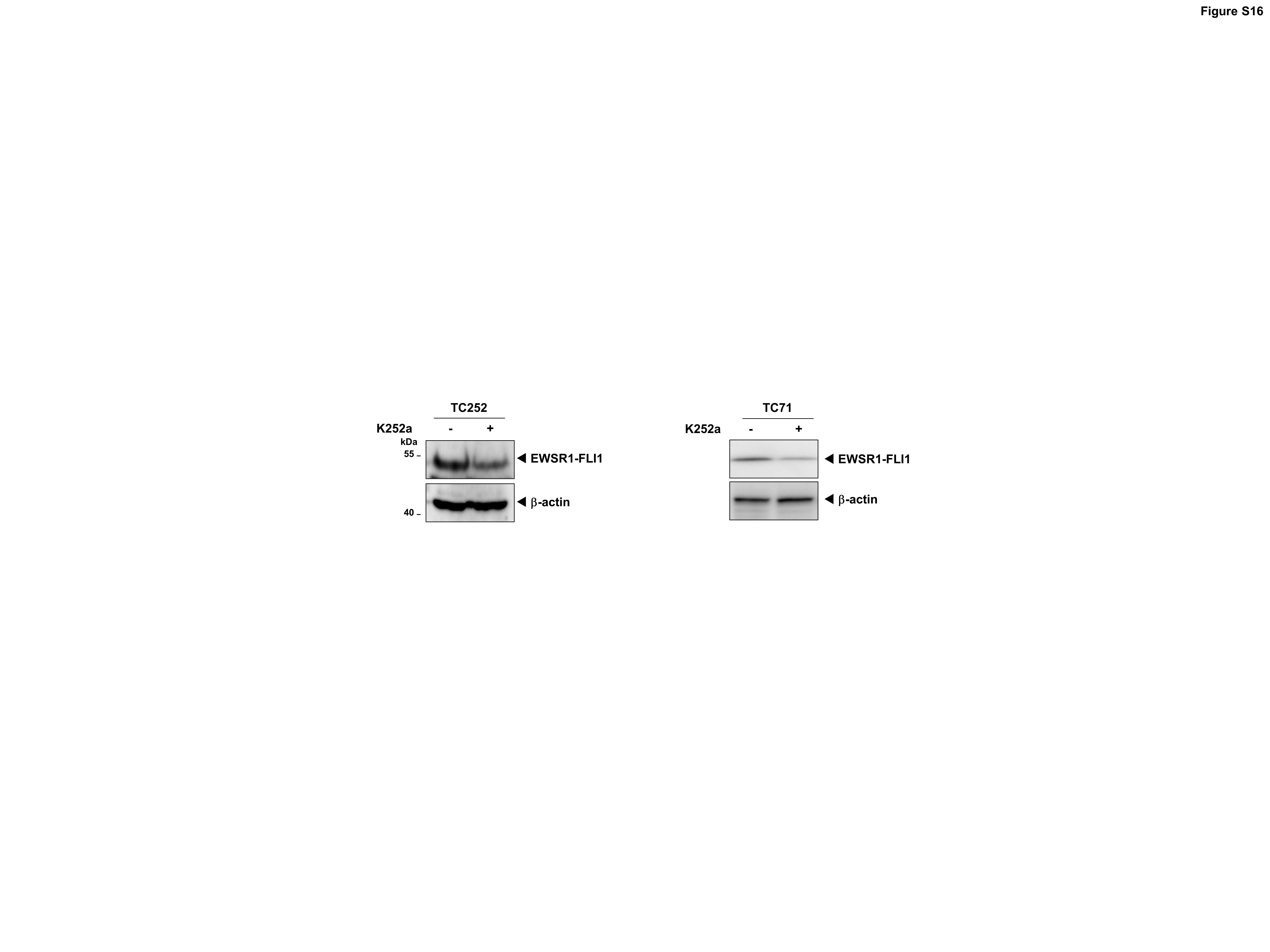

Supplement: Supplementary file 19 — Supplementary Figure 16 [file 41419_2022_5275_MOESM19_ESM.jpg]

**Fig. 3A**

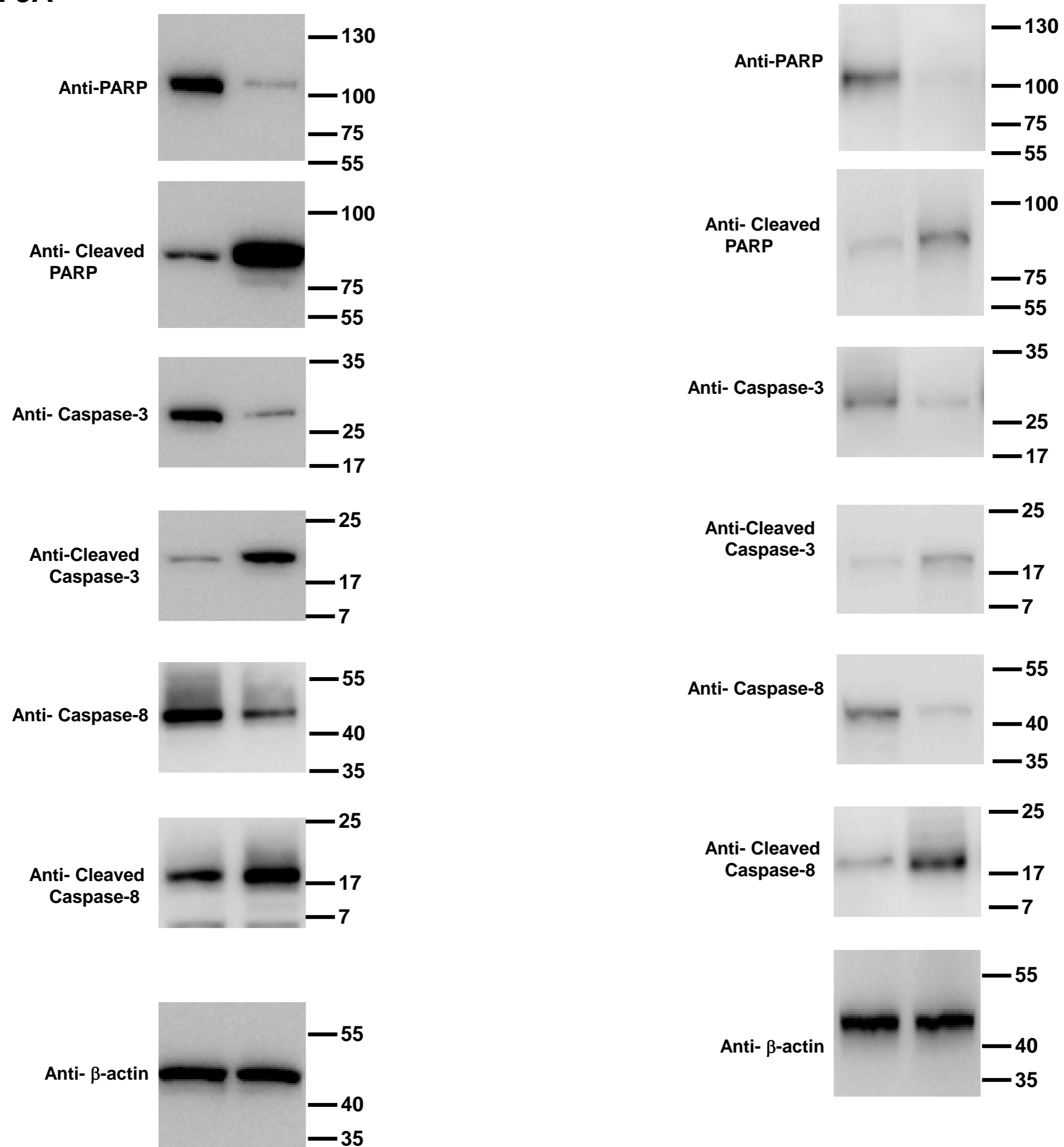

**Fig. 3E**

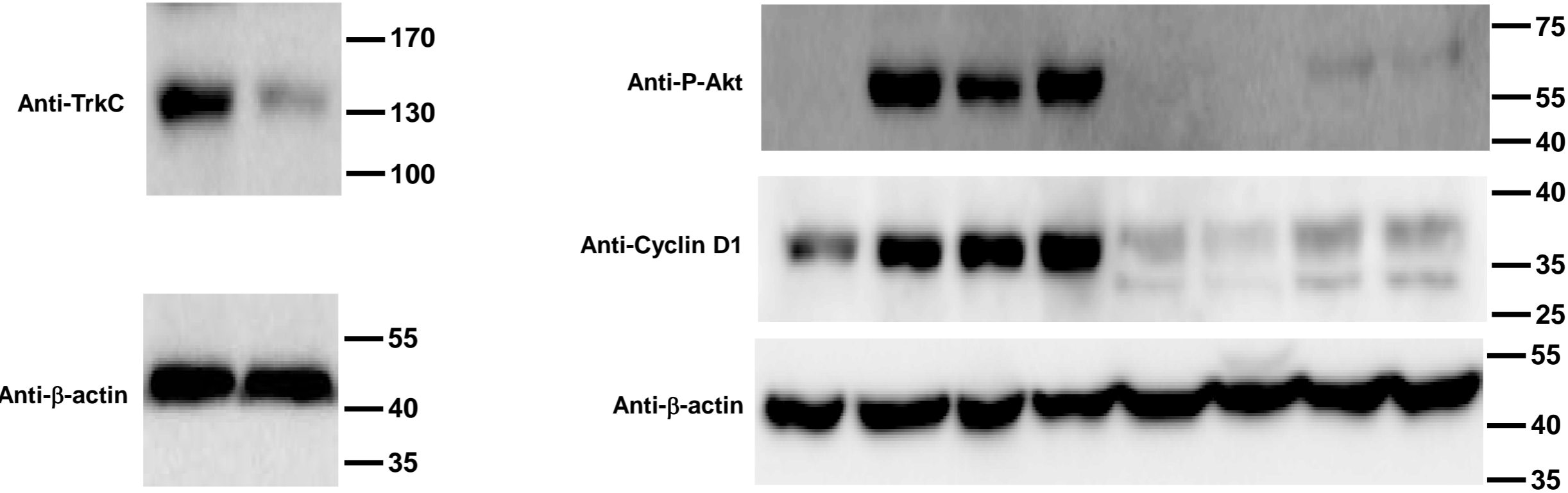

**Fig. 4F**

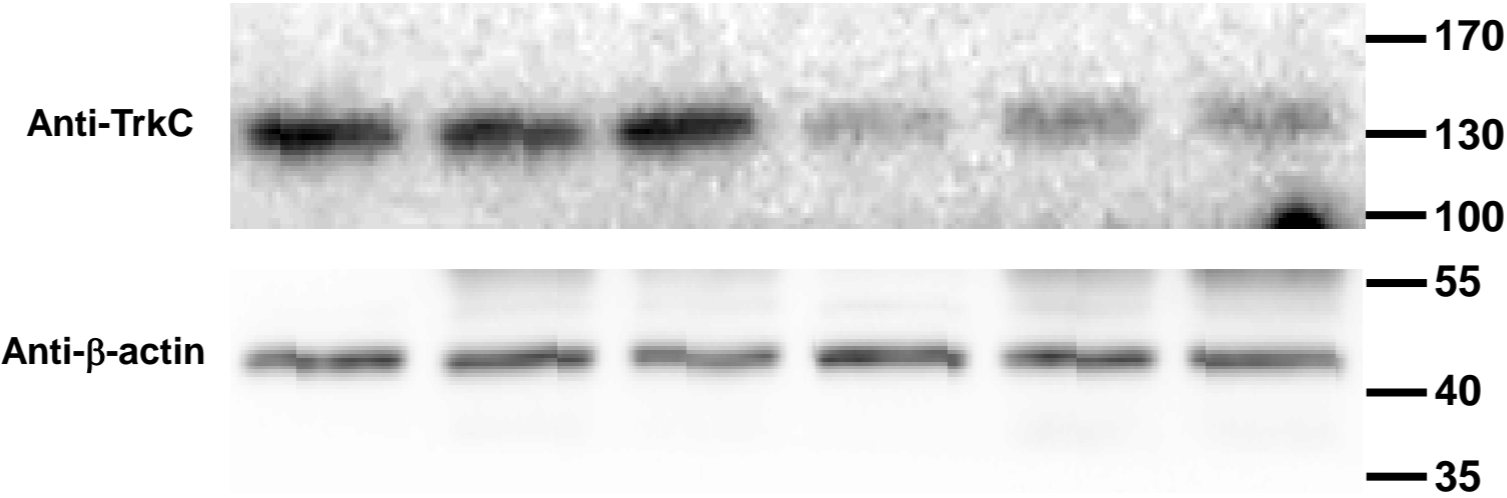

**Fig. 5B**

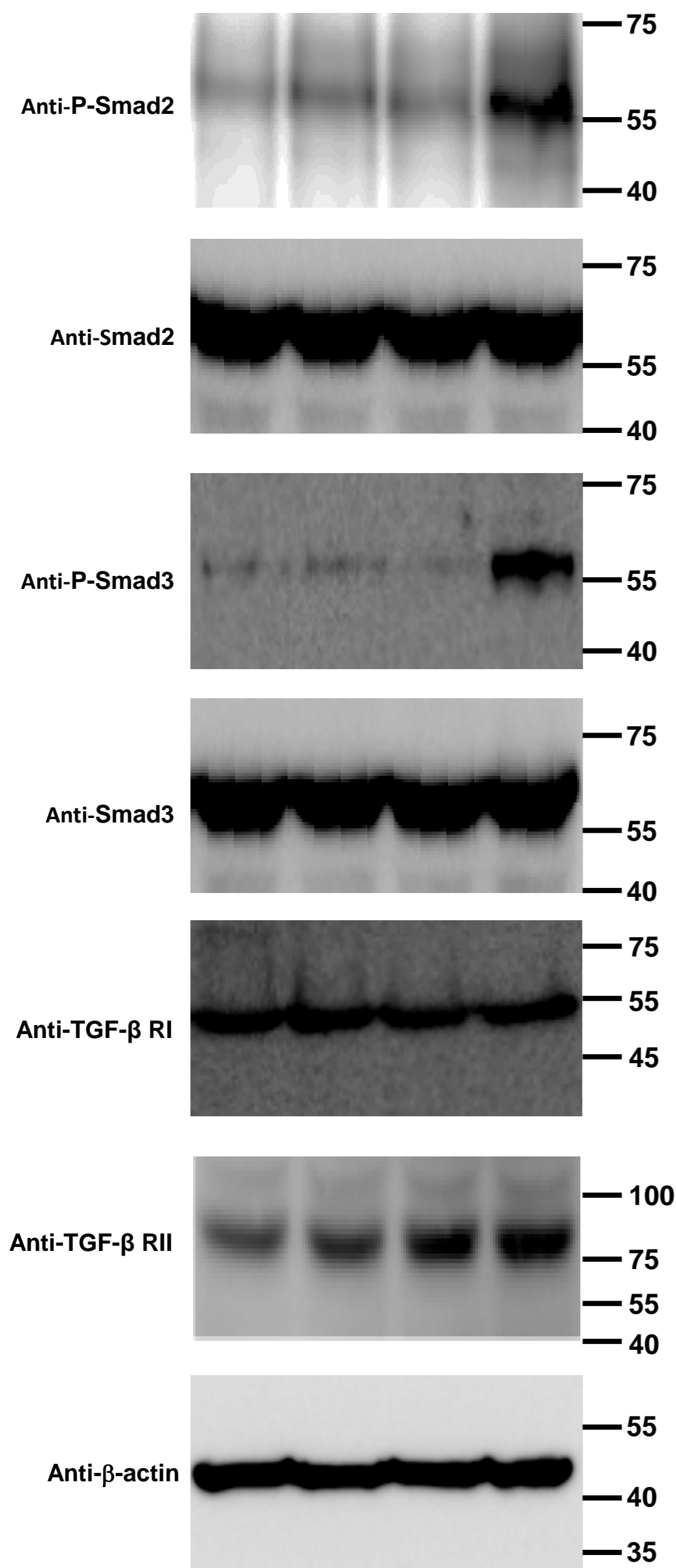

**Fig. 5C**

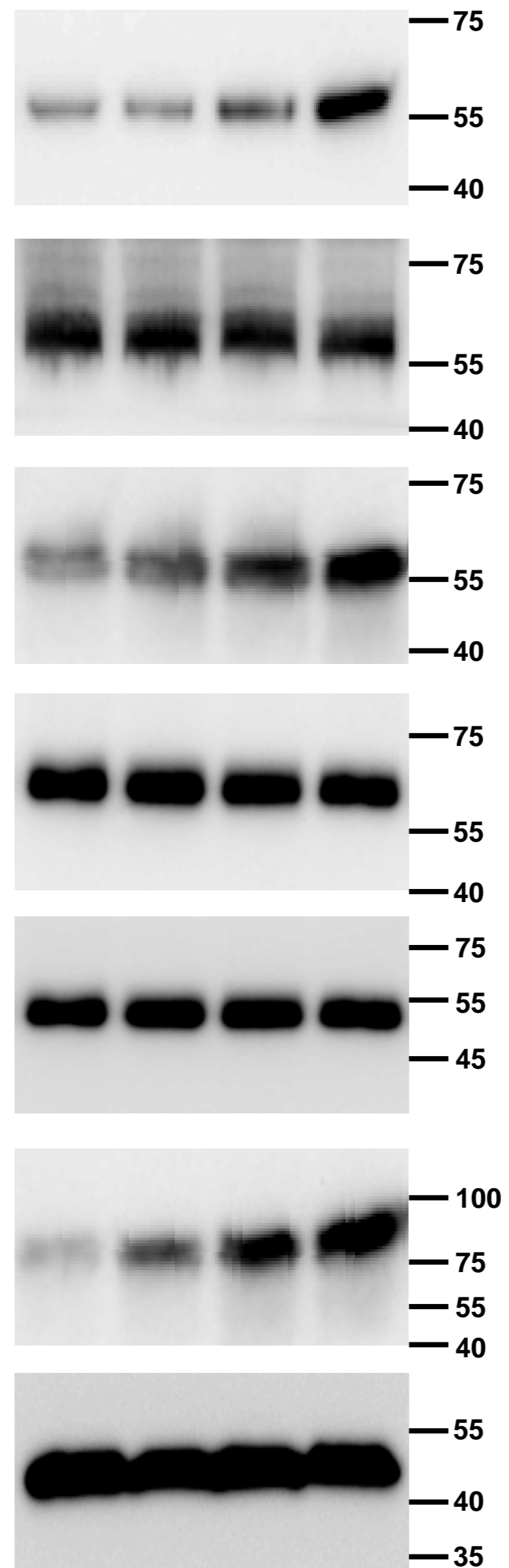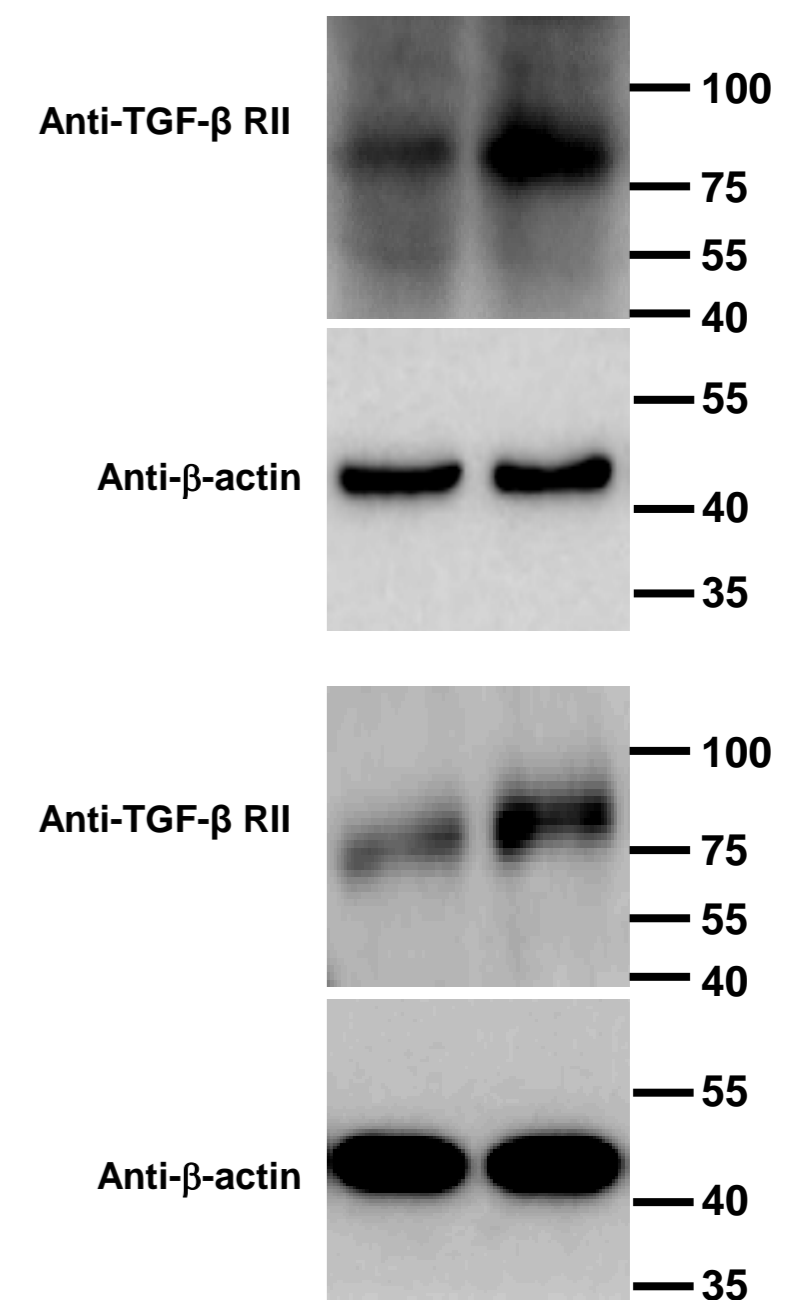

**Fig. 6A**

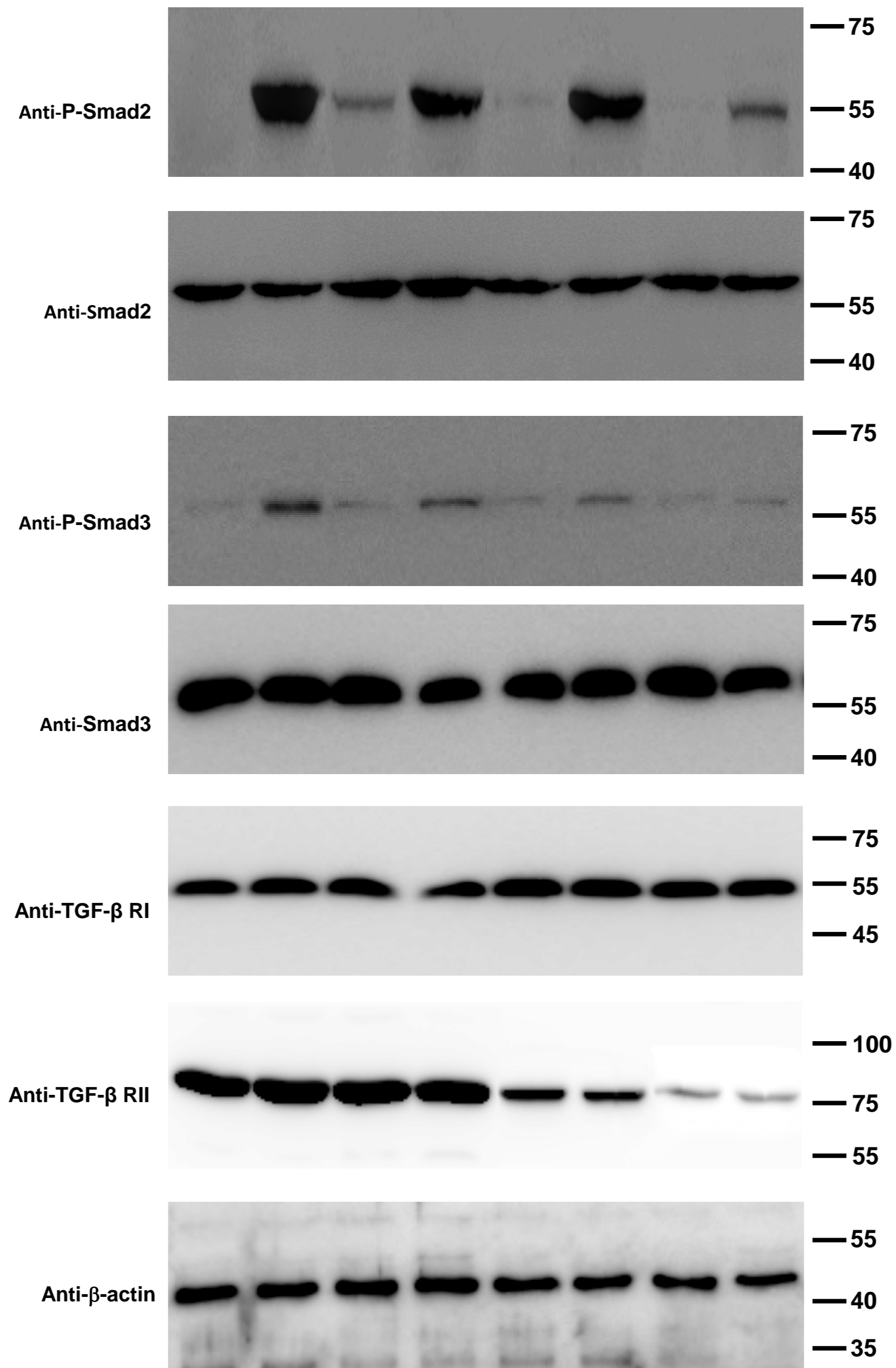

**Fig. 7A**

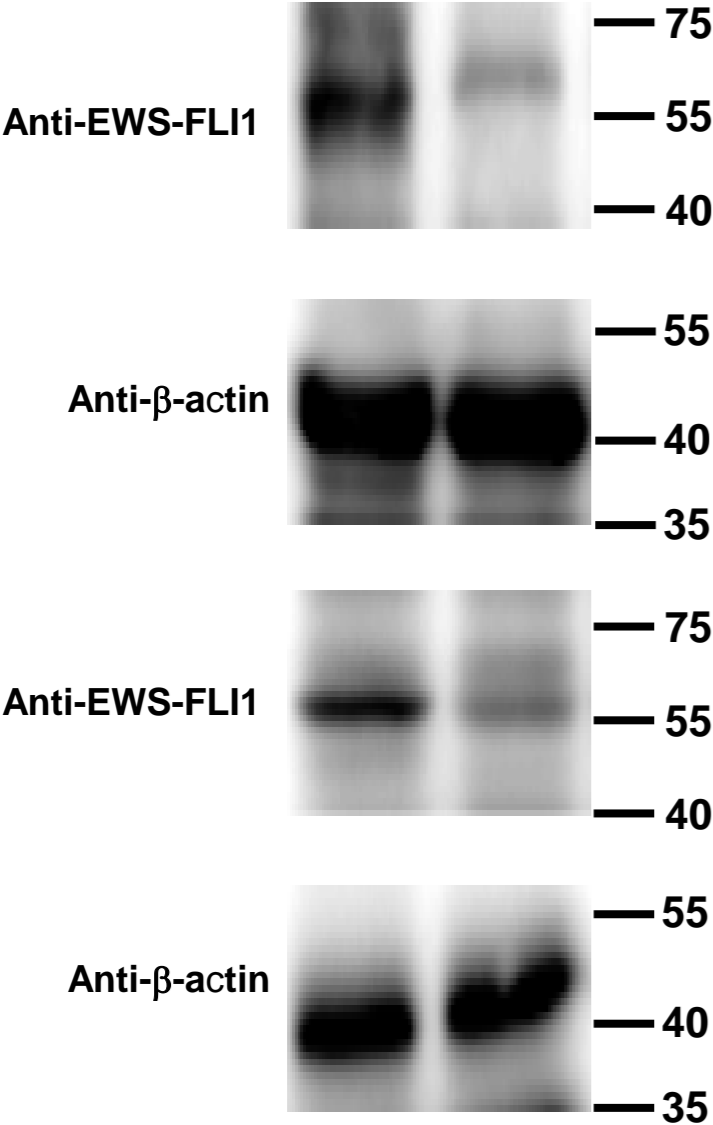

**Fig. 7D**

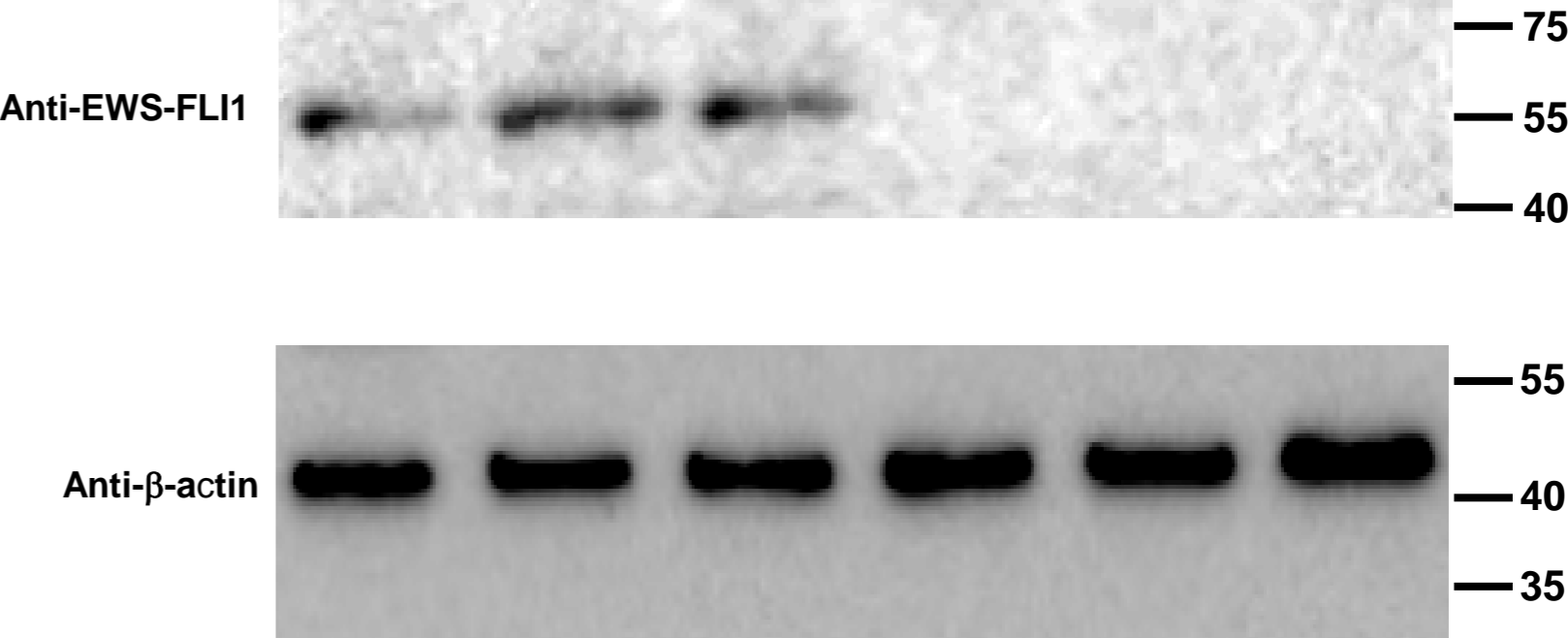

**Fig. 7E**

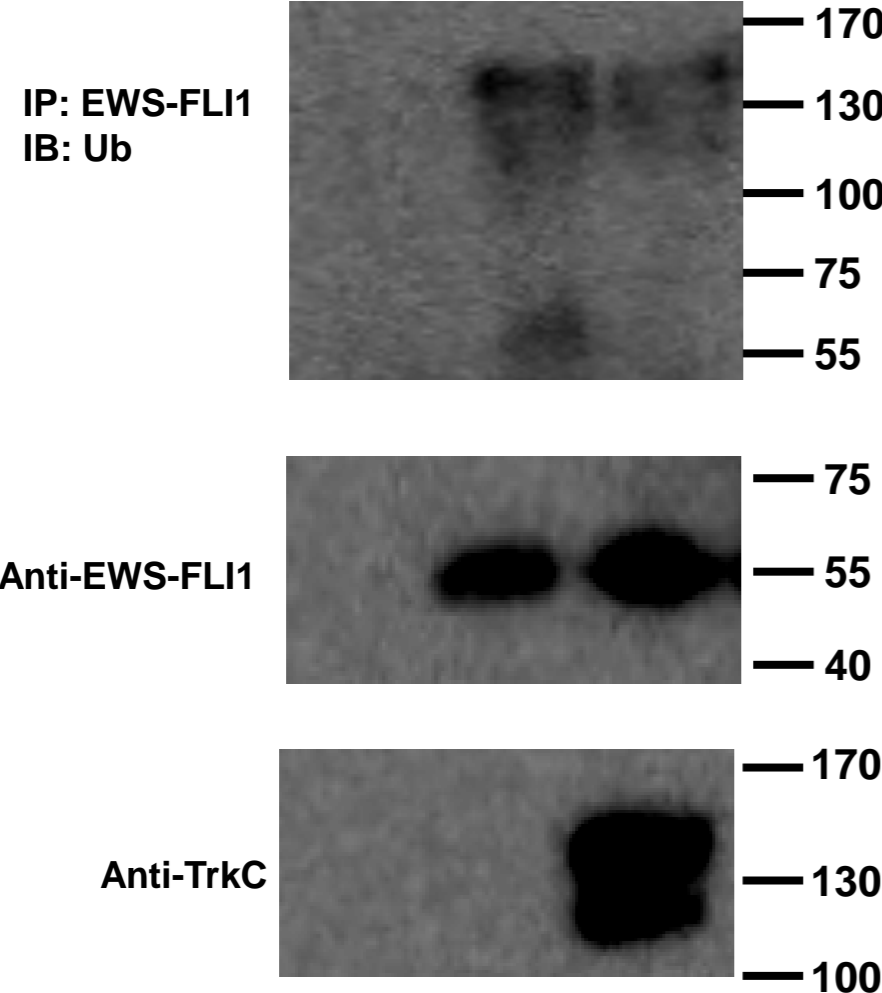

**Fig. 7F**

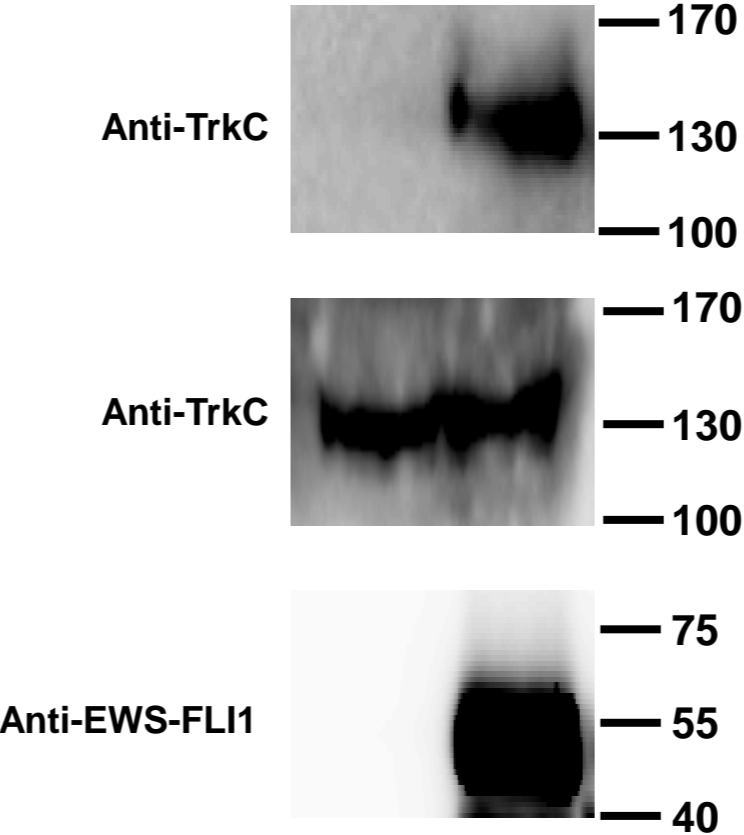

**Fig. 7G**

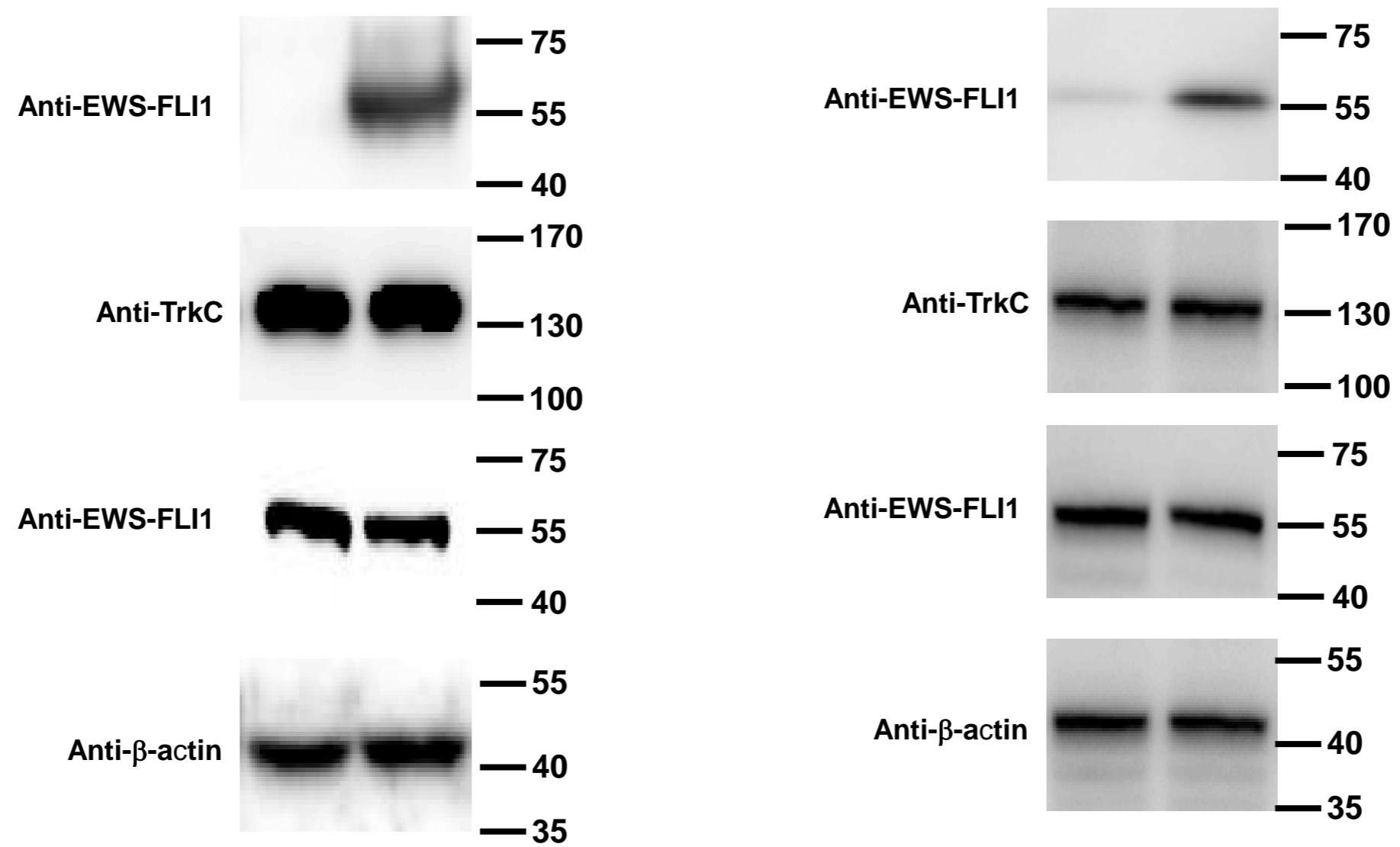

**Fig. 7H**

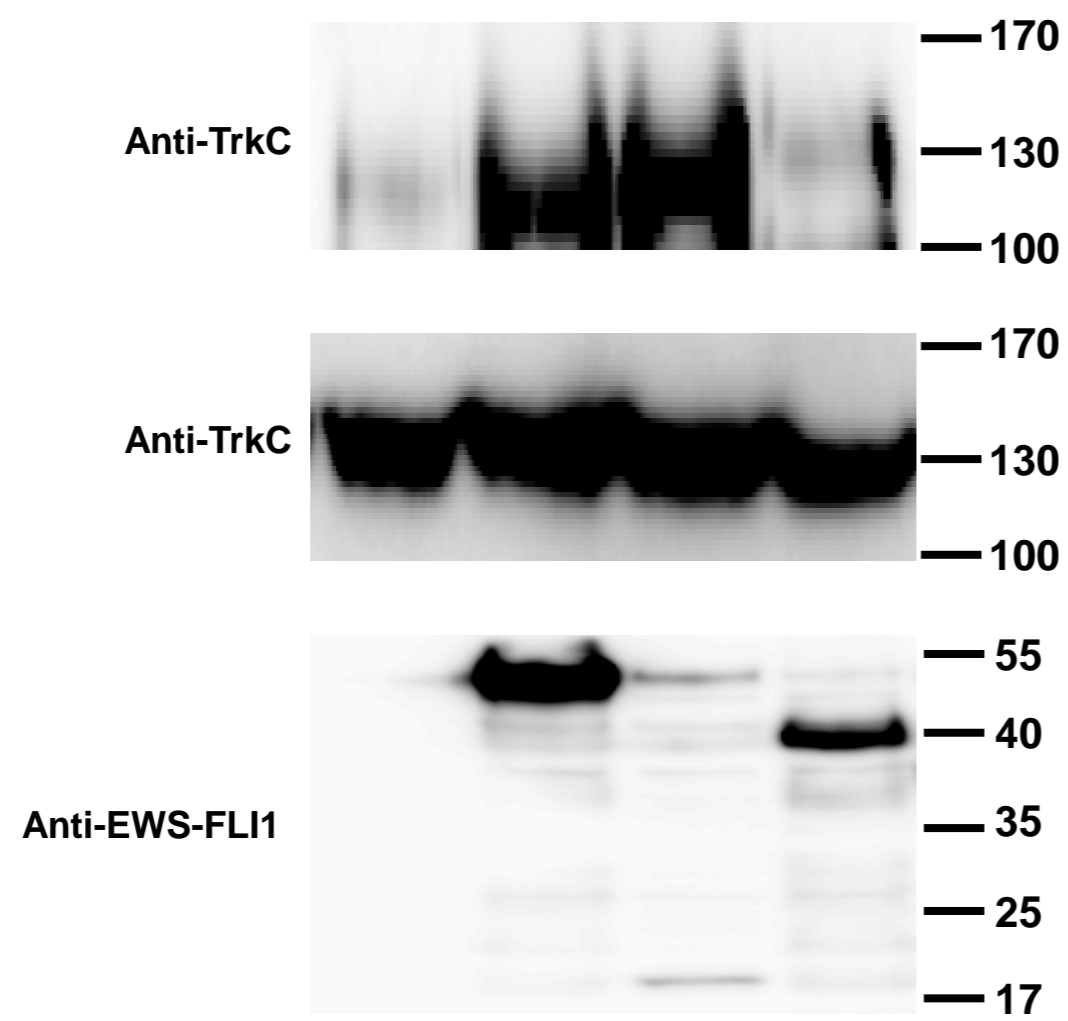

Supplement: Supplementary file 20 — Original western blots [file 41419_2022_5275_MOESM20_ESM.pdf]
